# Accounting for regional transmission variability and the impact of malaria control interventions in Ghana: a population level mathematical modelling approach

**DOI:** 10.1186/s12936-020-03496-y

**Published:** 2020-11-23

**Authors:** Timothy Awine, Sheetal P. Silal

**Affiliations:** 1 Modelling and Simulation Hub, Africa, Department of Statistical Sciences, University of Cape Town, Cape Town, South Africa; 2 South African Department of Science and Technology/National Research Foundation Centre of Excellence in Epidemiological Modelling and Analysis (SACEMA), University of Stellenbosch, Stellenbosch, South Africa; 3 Honorary Visiting Research Fellow in Tropical Disease Modelling, Nuffield Department of Medicine, University of Oxford, Oxford, UK

**Keywords:** Model, Malaria, Interventions, Long lasting insecticide bednets, Indoor residual spraying

## Abstract

**Background:**

This paper investigates the impact of malaria preventive interventions in Ghana and the prospects of achieving programme goals using mathematical models based on regionally diverse climatic zones of the country.

**Methods:**

Using data from the District Health Information Management System of the Ghana Health Service from 2008 to 2017, and historical intervention coverage levels, ordinary non-linear differential equations models were developed. These models incorporated transitions amongst various disease compartments for the three main ecological zones in Ghana. The Approximate Bayesian Computational sampling approach, with a distance based rejection criteria, was adopted for calibration. A leave-one-out approach was used to validate model parameters and the most sensitive parameters were evaluated using a multivariate regression analysis. The impact of insecticide-treated bed nets and their usage, and indoor residual spraying, as well as their protective efficacy on the incidence of malaria, was simulated at various levels of coverage and protective effectiveness in each ecological zone to investigate the prospects of achieving goals of the Ghana malaria control strategy for 2014–2020.

**Results:**

Increasing the coverage levels of both long-lasting insecticide-treated bed nets and indoor residual spraying activities, without a corresponding increase in their recommended utilization, does not impact highly on averting predicted incidence of malaria. Improving proper usage of long-lasting insecticide-treated bed nets could lead to substantial reductions in the predicted incidence of malaria. Similar results were obtained with indoor residual spraying across all ecological zones of Ghana.

**Conclusions:**

Projected goals set in the national strategic plan for malaria control 2014–2020, as well as World Health Organization targets for malaria pre-elimination by 2030, are only likely to be achieved if a substantial improvement in treated bed net usage is achieved, coupled with targeted deployment of indoor residual spraying with high community acceptability and efficacy.

## Background

Many malaria endemic countries, including Ghana, are making tremendous efforts aimed at achieving the 2016–2030 agenda towards malaria control and elimination [1, 2]. In line with this, the Ghana National Malaria Control Programme (NMCP) is guided by a national malaria strategic plan to reduce the burden of malaria by 75.0% across the country by 2020. Among the key strategic interventions adopted by the NMCP for achieving this milestone is the scaling of insecticide-treated bed nets (ITNs)/long-lasting insecticide-treated bed nets (LLINs) distribution, targeted indoor residual spraying (IRS), and improving monitoring activities [3, 4].

In recent years, the NMCP, with support from partners, such as the United States Agency for International Development (USAID), President’s Malaria Initiative (PMI) and The Global Fund to Fight AIDS, Tuberculosis and Malaria, have achieved considerable reductions in malaria-related mortalities, but progress towards substantial reductions in morbidity still remains a challenge [5]. These achievements follow the deployment of new intervention strategies following the adoption of new national policies on the use of the following:

- artemisinin-based combination therapy (ACT) as first line therapies for uncomplicated malaria between 2002 and 2004,
- scale up and distribution of ITNs in 2002 and thereafter,
- intermittent preventive treatment of malaria in pregnancy (IPTp) using sulfadoxine-pyrimethamine (SP) between 2003–2004,
- and IRS on a small scale in 2005 [5, 6].

Although these interventions are in place, evaluating their effectiveness using mechanistic models based on locally available data still remains largely unexplored [6]. Despite the contributions of earlier developed mathematical models’ describing the transmission dynamics of malaria in the country, there still exist important knowledge gaps in determining a rational basis for deploying these interventions and evaluating them in the three different ecological zones of Ghana [7].

The dynamics of malaria morbidity generally follow patterns of ecological factors such as rainfall and temperature [8]. There is evidence supporting both this spatial heterogeneity in the ecology of Ghana and the burden of malaria. For this reason, the spatial scale should not be ignored in any malaria investigations of national scale. The country was, therefore, partitioned into zones along three main ecological zones of Ghana, namely the Guinea savannah, transitional forest, and coastal savannah, as described elsewhere [8]. The model was then fitted to data for each zone.

Examples abound of uses of compartmental models for investigation of diseases with the aim of understanding the underlying principles or processes governing dynamics of diseases [9]. Since their introduction into public health by Bernoulli in 1766, applications of mathematical models focused on malaria transmission have continued to attract interest, with several models developed especially in the last fifty years. These models build on those formulated by Ross and vary in complexity and diversity, specifically to elucidate further understanding into the mechanism of malaria transmission in humans [10–13]. Currently, mathematical models are also being used, among others, to support the formulation of policies aimed at controlling diseases, including monitoring and evaluation of disease incidence [14].

The model developed in this study is based on the basic susceptible-infected-recovered-susceptible (SIRS) model [15, 16], which has been modified to include additional compartments and attributes of the transmission settings in Ghana, such as superinfection. The model structure includes a human population model coupled with a vector model, with climatic elements adapted from Agusto et al*.* [17].

The objective of this paper is to develop a mathematical model to project the impact of various intervention scenarios of malaria intervention control programmes in Ghana, simulated at a sub-population level that represents the three main ecological zones [7]. The impact of various levels of usage and protective effectiveness, as well as coverage of LLINs and IRS, is also investigated, and prospects of achieving relevant locally and internationally set goals of malaria control and elimination in Ghana are considered.

## Methods

**Fig. 1:**
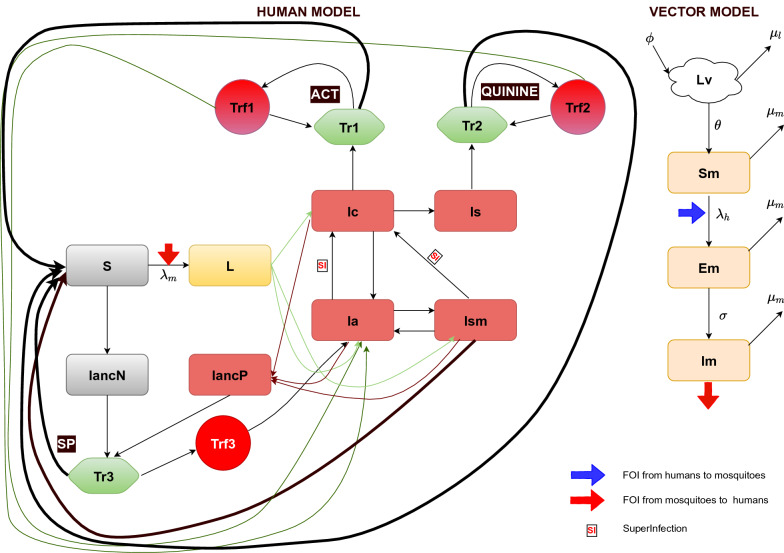
Malaria transmission model showing various compartments of both human and vector populations

Ordinary differential equations were used to develop compartmental models for malaria transmission dynamics in the three ecological zones of Ghana. The model diagram for both human and vector populations is as illustrated in Fig. 1. Further details and a description of the models are presented in Additional file 1: S1 Text, the online supplementary files.

### Model structure

*Human population* **S** represents the susceptible human compartment (where different probabilities have been applied, respectively, to recruited naïve or non-immuned children under 6 years of age, children 6 and above, and adults and pregnant women into the latent stage **L** before the onset of gametocytes. The **Ic**, **Ia**, **Is** and **Ism** compartments represent symptomatic infection (clinical infection), asymptomatic infection, severe infection and sub-microscopic infection, respectively. Pregnant women attend antenatal clinic (ANC) without an infection in **IANCN** or progress from **L3** into **IANCP** once infected. **Tr1**, **Tr2,** and **Tr3** represent the treatment sought for confirmed uncomplicated malaria (**Ic**), severe malaria (**Is**), and routine monthly SP prophylaxis for pregnant women at ANC, respectively. **Trf1**, **Trf2** and **Trf3** represent respective treatment failure due to adherence and possible drug resistance to the aforementioned three treatment options.

*Vector population* **Lv** represents the larvae population and **Sm** the susceptible mosquitoes. Exposed mosquitoes are captured in the **Em** compartment, whereas infectious mosquitoes are found in the **Im** compartment. The grey compartments represent the populations which are susceptible, yellow those with latent infection, brown those with a blood stage infection and green those with symptomatic infection and which undergo treatment. Compartments for treatment failure are indicated in red. The red and blue arrows present the forces of infection from infectious mosquitoes to humans and infectious humans to mosquitoes, respectively.

The model diagram shown in Fig. 1 above depicts a vector-coupled malaria transmission model that includes compartments for various stages of malaria and subsections of the Ghanaian population. The subsections of the population captured are children ≥ 6 years and adults, children under 6 years, and pregnant women, even though the models are not age structured.

The stages of development of the malaria parasite and the mosquito are captured by four compartments representing the young and adult mosquitoes, which can be classified as being susceptible, infected, and infectious, once ingested parasite(s) complete the full cycle of development.

### Source of clinical data

Confirmed cases of uncomplicated, severe malaria and malaria in pregnancy reported by health facilities spanning 2008 to 2017 were used. Data for each zone consist of aggregated monthly caseloads for regions of the Guinea savannah (Upper East, Upper West and Northern regions), transitional forest (Ashanti, Brong-Ahafo, Eastern and Volta regions) and coastal savannah (Central, Greater Accra and Western regions) [8]. The health facilities where data were captured are located in all 216 districts across all regions of Ghana. The parameters used were sourced from literature or from the data fitting process to account for zonal transmission diversity. This was done to capture the different dynamics of morbidity of malaria and to allow for a better evaluation of the effectiveness of the various interventions in these zones.

**Fig. 2:**
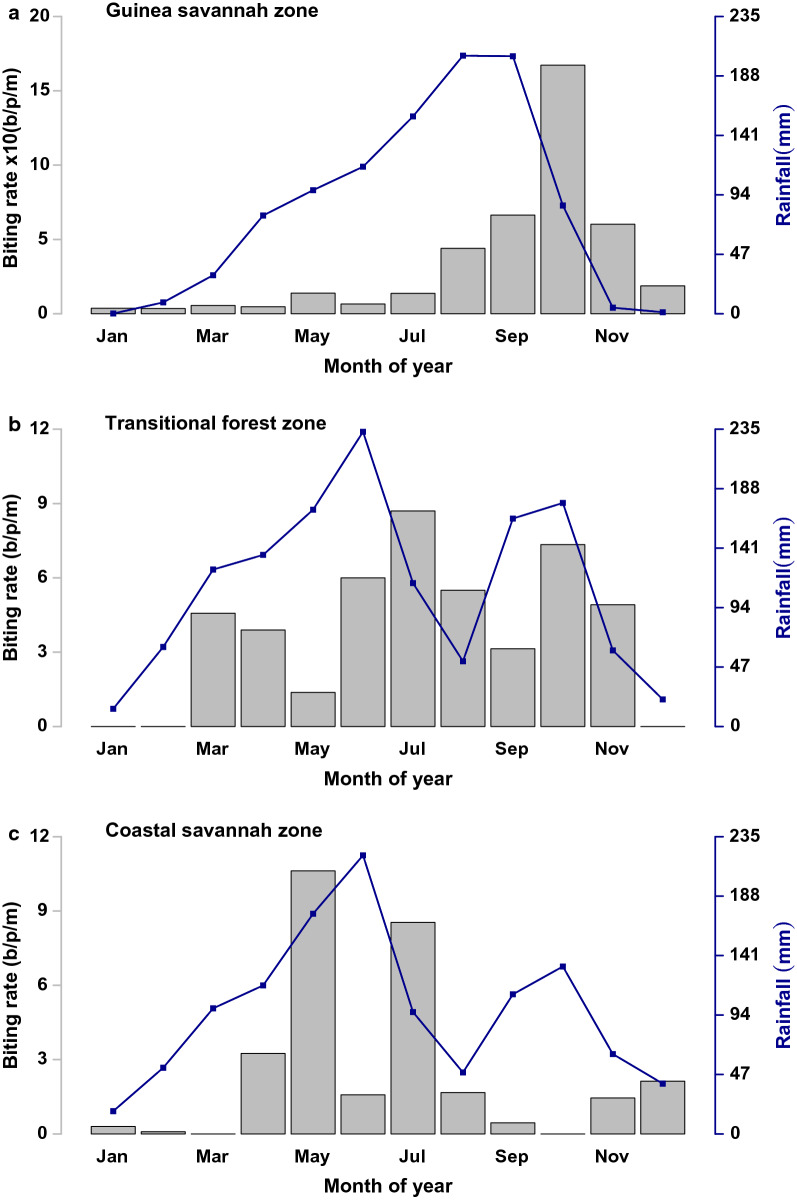
Monthly biting rates (b/p/m) [Grey Bars] and rainfall (mm) [Blue Lines] in the Guinea savannah, Transitional forest and Coastal savannah, respectively

While these parameters are captured in the table of parameters as shown in Table 1, reported biting rates of humans by mosquitoes used for the data fitting are shown in Fig. 2. The reported uncomplicated malaria data used in this study are depicted under Baseline of the results section.

**Table 1:** Parameter values

Parameter name	Parameter value by Zone					Parameter definition	Source
	Guinea Savannah	Transitional Forest		Coastal Savannah			
pc1	0.90	0.90		0.80		Probability of naive progressing into **Ic**	Estimated
pa1	0.35	0.07		0.58		Probability of naive progressing into **Ia**	Estimated
pc2	0.14	0.19		0.14		Probability of non-naive progressing into **Ic**	Estimated
pa2	0.61	0.39		0.49		Probability of non-naive progressing into **Ia**	Estimated
Ps	0.130	0.065		0.062		Probability of progressing into severe disease	[52]
pt1	0.87	0.88		0.88		Probability of being tested/diagnosed for uncomplicated malaria	[53]
Ppc	0.81	0.70		0.80		Proportion of pregnant women from **L3** progressing to **Ic**	Estimated
Ppa	0.250	0.075		0.540		Proportion of pregnant women from **L3** progressing to **Ia**	Estimated
X	0.01	0.01		0.01		Probability of progressing from **Ia** to **Ic**	Estimated
m1	0.57	0.10		0.10		Probability of infection among children under 6 years and pregnant women	Estimated
m2	0.77	0.22		0.20		Probability of infection among non-naive population 6 years and above	Estimated
Pst	0.80	0.71		0.73		Probability of seeking treatment at the health facility	[53]
Prob_inf	0.50					Probability of a bite resulting into a mosquito being infected or a human being infected following a bite from an mosquito	[15]
Pn	0.125	0.125		0.125		Proportion of population of children under 6 years	[54]
Pm	0.874375	0.87425		0.8744125		Proportion of population 6 years and above	[15]
pt2	0.99	0.99		0.99		Probability of being treated with **QUININE**	[55]
ah1	0.385	0.385		0.385		Proportion non-adherent to **ACT** treatment	[56]
ah2	0.092	0.082		0.082		Proportion non-adherent to **QUININE** treatment	[55, 57]
Px	0.025					Proportion of pregnant women in the population	[54]
rs1	0.04	0.04		0.04		Resistance against **ACT** (day 28 PCR-corrected failure rate (0.8–4.0%) for ASAQ and AL	[58]
rs2	0.01	0.01		0.01		Resistance against **QUININE,** intramusclar **ARTEMETHER** (day 28 parasitaemia failure rate)	[59]
rs3	0.0962	0.0962		0.0962		Resistance against **SP** (day 28 PCR-corrected failure rate (0.0962) for SP	[60]
ac1	0.134	0.126		0.112		Probability of asymptomatic malaria among pregnant women at **ANC**	[61–63]
ac2	0.097	0.097		0.097		Probability of sub-microscopic infection among pregnant women at **ANC**	[54]
pt3	0.367					Proportion of pregnant women taking up at least 3 dose	[64]
ah3	0.633					Proportion of pregnant women not taking up at least 3 doses	[64]
Nn	5.1 × 10^6^	17.1 × 10^6^		8.1 × 10^6^		Human population size (2018 mid-year estimated) (number)	DHIMS2
Ln	25	30.6		23.5		birth/death rate per 1000 population (year^−1^)	[65–67]
Kv	7.8 × 10^5^	4.2 × 10^7^		2.5 × 10^7^		Carrying capacity of the environment for larva and pupae stages of mosquitoes (ha^−1^)	estimated
LLIN	0.398					Protective efficacy of **LLINs** against malaria (based on the IRR^a^ or OR^b^)	[31]
IRS	0.285					Protective efficacy of **IRS** against malaria (based on the IRR^a^ or OR^b^)	[31]
Ss	365.25/5					Rate of progressing into severe disease (day^−1^)	[68]
Q	365.25/194					Duration of progressing from **Ia** into **Ic** (day^−1^)	[33, 56, 69]
gamma	365.25/21					Duration of latent period in human population (day^−1^)	[70]
t1	365.25/3					Duration after onset of illness **ACT** treatment was sought (day^−1^)	[70]
rho1	365.25/3					Recovery rate after ACT treatment (day^−1^)	[70, 71]
rho2	365.25/6					Recovery rate after **QUININE** treatment (day^−1^)	[72]
V	52/5.5					Rate of recovery from **Ia** to **Ism** without treatment (week^−1^)	[73]
Nr	365.25/130					Rate of natural recovery from infection (day^−1^)	[74]
AC	365.25/30					Rate of antenatal attendance (day^−1^)	[75]
Hlsp	365.25/8					Rate of recovering after **SP** treatment at **ANC** (day^−1^)	[76]
RDTMicSens	0.49					Average sensitivity of **RDTs** and Microscopy in health facilities (proportion)	[77]
Reporting	0.969		0.966		0.947	Reporting probability of uncomplicated malaria at health facility (proportion)	Data from NMCP

^a^*IRR* incidence rate ratio

^b^*OR* Odds ratio

The most populous of the zones is the transitional forest zone, with a population of 17.1 million, followed by the coastal savannah zone with 8.1 million, while the Guinea savannah zone accounts for 5.1 million people (using 2017 zonal estimated population from DHIMS2).

### Force of infection

Transmission of malaria parasites between humans and mosquitoes is through the drawing of blood from humans by infectious mosquitoes. The likelihood of humans’ being infected upon a successful bite from a mosquito will to some extent depend on the level of susceptibility of getting infected. On the other hand, a non-infected mosquito which draws blood from an infected human also has a probability of ingesting gametocytes which later develop into sporozoites.

In these models, a 50.0% chance (prob_inf) of transmitting the malaria parasite between humans and mosquitoes following a successful bite of an infected mosquito on humans, or an uninfected mosquito on humans in any of the infected stages, was considered [15].

Transmission is governed by the forces of infection (**λ**_**mh**_ and **λ**_**hm**_) from mosquitoes to humans and human to mosquitoes, respectively.

The forces of infections are defined as:

1 $${{\varvec{\uplambda}}}_{{{\mathbf{hm}}}} = {\text{ prob}}\_{\text{inf}} \times \left( {1 - {\text{ ITNcov}} \times {\text{ITNeff}} \times {\text{ITNusage}}} \right) \times \left( {1 - {\text{ IRScov}} \times {\text{IRSeff}}} \right) \times {\text{BR}} \times \frac{{\left( {{\text{Ic}}_{{\text{h}}} + {\text{Ia}}_{{\text{h}}} + {\text{Isd}}_{{\text{h}}} + {\text{Ism}}_{{\text{h}}} + {\text{IancP}}_{{\text{h}}} + {\text{Tr}}1_{{\text{h}}} + {\text{Tr}}2_{{\text{h}}} + {\text{Tr}}3_{{\text{h}}} + {\text{Trf}}1_{{\text{h}}} + {\text{Trf}}2_{{\text{h}}} + {\text{Trf}}3_{{\text{h}}} } \right)}}{{{\text{N}}_{{\text{h}}} }}$$

2 $${{\varvec{\uplambda}}}_{{{\mathbf{mh}}}} = {\text{prob}}\_{\text{inf}} \times \left( {1 - {\text{ ITNcov}} \times {\text{ITNeff}} \times {\text{ITNusage}}} \right) \times \left( {1 - {\text{ IRScov}} \times {\text{IRSeff}}} \right) \times {\text{BR}} \times \frac{{{\text{I}}_{{\text{m}}} }}{{{\text{N}}_{{\text{h}}} }}$$

Equation (1) represents the force of infection from humans to mosquitoes and Eq. (2) represents the force of infection from mosquitoes to humans. The contact rate is represented by the biting rate (BR). The BR data were obtained from field studies from each of the zones through human landing catches (HLC). They are defined as the average number of bites received by a human in the population per month (b/p/m), as shown in Fig. 2 [18, 19].

As captured in Fig. 2a, biting rates in the Guinea savannah could be as high as 170 (b/p/m), whereas those of the Transitional forest were 12 (b/p/m) and 10 (b/p/m) in the Coastal savannah during the peak transmission seasons respectively. Figure 2 suggests that even though biting (as well as transmission) seems to occur all year around, in all zones, the peak follows rising rainfall.

Levels of coverage, usage and effectiveness of ITNs and IRS are denoted by *itnc*(*t*) = (1 − *itncov* × *itnusage* × *itneff*) and *irsc*(*t*) = (1 − *irscov* × *irseff*) respectively, where *itncov* and *irscov* and *itneff* and *irseff* represent coverage levels and effectiveness for ITNs and IRS respectively, with *t* being time steps and *itnusage* the level of ITN/LLIN usage.

### Immunity and superinfection

The stable nature of transmission and the variation in seasonality across all three ecological zones requires incorporating superinfection, acquired immunity, treatment failure, and seasonality into the model structure. This is to account for the natural history of malaria as much as possible, which allows for the description of the transmission dynamics of malaria across Ghana.

The models do not incorporate levels of immunity following length of exposure based on age (two broad age classifications for children under 5 and adults); however, aspects of the model structure account for this concept, though not fully. Thus the transitions accounting for some level of immunity in the model are:

- Children born naïve or young children with little exposure to malaria infection:
- born into the **Sn** compartment or
- born with a congenital infection of malaria into the **Ia** compartment
- Adults in the population with several years of exposure
- recruited into the Snn compartment

- Progressing from Ism to Ic
- Progressing from Ic to Ia
- Progressing from Ia to Ism
- Recovering naturally without treatment from Ism to Snn [20]; a state of susceptibility where one is more likely to have an asymptomatic episode of malaria.

Reinfection or superinfection was allowed in the models given the high transmission settings of all zones across Ghana. A factor that is dependent on the inverse of the sum of the rate of force of infection from mosquito to human and duration of infection, i.e. (1/${\lambda }_{m}$+1/$\gamma$)^−1^_,_ was incorporated. This factor affects the populations in the infected compartments **Ia** and **Ism**. A proportion of the infected and superinfected therefore make the following transitions:

- Progressing from Ia to Ic
- Progressing from Ism to Ic

### Vector dynamics

From Fig. 1, the vector compartments **Lv**, **Sm**, **Em,** and **Im** respectively represent young mosquitoes (larvae and pupae), susceptible mosquitoes, exposed mosquitoes, and infectious mosquitoes. The compartment for susceptible mosquitoes is populated from the maturing larvae and pupae compartment, **Lv**. The egg deposition rate, $\boldsymbol{\varnothing }$, and maturing rate, ***θ***, are both dependent on the carrying capacity (**Kv**) of the environment to support breeding, which in turn depends on rainfall (R_f_) and environmental temperature (Temp). The incorporation of these environmental factors, as shown in Fig. 2, drives the transmission dynamics of malaria incidence in the various zones, which generally lags behind seasonal rainfall [8]. Details of the governing equations of the Vector model can be found in the Additional file 1: S1 text file.

### Data fitting

**Fig. 3:**
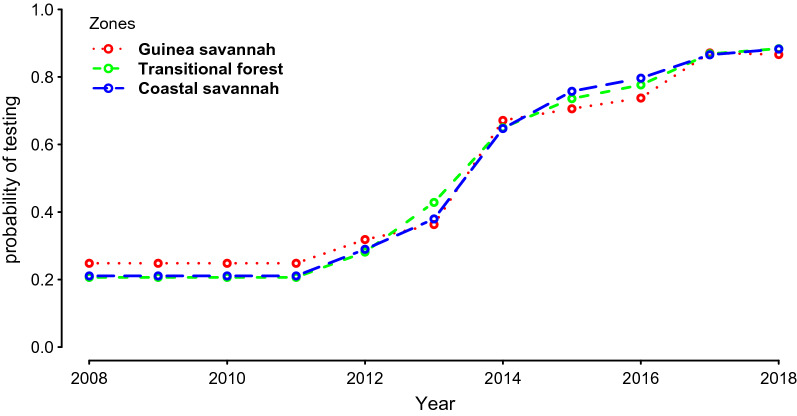
Probability of testing all suspected malaria cases by zone (Source: NMCP).

Zonal-specific monthly confirmed reported uncomplicated malaria cases, severe malaria cases, and malaria among pregnant women, were used for data fitting after the models attained steady state. With regards to data fitting, data from 2008 to 2017 on the DHIMS were used. The observed rising trend of malaria cases for this period seems to suggest an increasing trend in the incidence of malaria in Ghana. However, as pointed out elsewhere, this seeming increasing trajectory is largely due to reporting, increasing diagnostic testing (Fig. 3), and potential improvement in health-seeking behaviour [8].

The models were first individually implemented from 1988 to 1997 to attain a steady state. They were then implemented across all zones from 1998 to 2017, incorporating reported levels of historical interventions, such as LLINs and IRS coverages. These were obtained from national surveys, such as Demographic and Health Surveys (DHS) and the Multiple Indicator Cluster Surveys (MICS), and annual reports of the NMCP. Historical Seasonal Malaria Chemotherapy (SMC) intervention coverage levels from 2015 were also incorporated in the data fitting stages of the model for the Guinea savannah zone (Additional file 1: S1 Fig. 2). These SMC coverage levels were obtained from reports of the NMCP, among others [21–24]. The data fitting phase was also adjusted for reporting probabilities of the health facilities’ capturing all confirmed cases of malaria onto the DHIMS platform. The probabilities of seeking treatment and receiving a diagnostic test at the health facility were all taken into account in the fitting process. The sources of these parameters are referenced in the table of parameters, Table 1.

In all 120 data points, each of three time series for uncomplicated malaria, severe malaria, and malaria in pregnancy were used for data fitting, as well as 10 estimated parameters as indicated in the table of parameters, Table 1. The incidence of confirmed malaria reported in 2017 was considered as baseline for future predictions. All the parameters from 2017 were then held constant from 2017 to 2030, which is the prediction period for scenario testing.

Data management was undertaken in Stata version 13.1 (StataCorp LP., College Station, Texas, USA). All analyses and computation were performed using R version 3.3.2 Copyright (C) 2018 [25].

### Model calibration

The dimensionality of the monthly aggregated counts of confirmed multiple categories of malaria cases from each ecological zone made direct parameter estimation through the computation of the likelihood difficult or intractable. The approximate Bayesian computation (ABC) approach was, therefore, deployed for model calibration.

Bayesian philosophy allows for the estimate of the posterior distribution of parameters to be computed using stochastic sampling of the prior parameter distribution. This process allows the calibration of parameters to be carried out, whilst avoiding the estimation of the likelihood function [26, 27]. ABC was implemented using a rejection criterion based on the Euclidean distance (Additional file 1: Eq. (6) of the S1 text) between summary statistics of predictions arising out of sampled parameter sets and summary statistics of observed monthly reported malaria cases in Ghana from 2008 to 2017 [28, 29]. Out of 15,000 iterations, 10–20% of the sample was retained for parameter validation.

The bands around the graphs in Figs. 5, 6 and 7 in the results section are 95% pseudo-confidence intervals. These were constructed for each month using 2.5% and 97.5% quantiles of the retained simulations.

### Parameter validation

A cross validation of the accuracy of the parameters was undertaken using the R package **cv4abc.** The sample parameters retained and used were based on a distance criterion between a summary statistic of the observed data and the simulated data, seen in Additional file 1: S1 Eq. (6)**.** A leave-one-out cross validation was implemented, and the prediction error for each parameter, as well as sensitivity or robustness to various tolerance levels, was calculated [30]. All simulations were performed on high performance computing facilities provided for by the ICTS High Performance Computing team (http://hpc.uct.ac.za) of the University of Cape Town.

### Sensitivity analyses

A multivariate regression-based sensitivity analysis of model parameters was performed for each zone. These investigations were carried out using the sample data obtained from the ABC analysis. The most sensitive parameters for each model were then obtained from an ordered set of standardized coefficients of parameters in the multivariate regression. Additional file 1: S1 text Tables 5, 6, and 7 show the most sensitive parameters by transmission zone.

### Interventions tested

In this study, the interventions investigated include the impact of elevated coverage (universal coverage defined as 1 treated bed net per 2 household members) and usage (proportion of the population reported to be sleeping under a treated bed net) levels, as well as protective effectiveness (PE) (proportion of cases of clinical malaria which could potentially be averted while using a treated bed net or dwelling in structures that have been sprayed with an approved insecticide to repel or kill mosquitoes) of ITNs or LLINs, and IRS.

Baseline LLIN and IRS average coverage levels in the various zones were 66.0%, 51.0%, 50.0% and 17.0%, 0.0%, 0.0%, respectively for the Guinea savannah, transitional forest, and coastal savannah zones. Additionally, LLIN usage at baseline was also 56.0%, 45.0% and 35.0% for Guinea savannah, transitional forest, and coastal savannah, respectively.

Various hypothetical scenarios were investigated with the aim of observing which ones resulted in the achievement of the targets set by the national malaria control strategic policy goals by fixed deadlines. The scenarios presented here include:

1. Implementation of only LLIN to achieve a universal coverage of 70.0% and 90.0% with usage at 60.0% within three years and IRS coverages at baseline across all zones.
2. Implementation of only IRS for a period of five years to achieve IRS coverage of 90.0% and PE of 30.0% and 60.0%, with LLIN coverage and usage at baseline levels (66.0% and 56.0% in the Guinea savannah, 51.0% and 45.0% in the transitional forest and 50.0% and 35.0% in the coastal savannah, respectively).
3. LLIN and IRS coverage at 80.0% and 80.0% versus 80.0% and 90.0%, respectively, maintaining LLIN usage at 60.0% and IRS PE at baseline (30.0% in the Guinea savannah, 30.0% in the transitional forest and 30.0% in the coastal savannah, respectively).

Other interventions tested but not presented here include the impact of SMC among children under 6 years in the Guinea savannah zone and Mass Screen and Treat (MSAT) in the transitional forest and coastal savannah zones.

Investigations carried out in this study were largely guided by the goals and objectives of the national malaria control strategic policy of 2014–2020. The findings have neither been approved by, nor were the recommendations arrived at made in consultation with, the NMCP in Ghana [4].

## Results

### Baseline

**Fig. 4:**
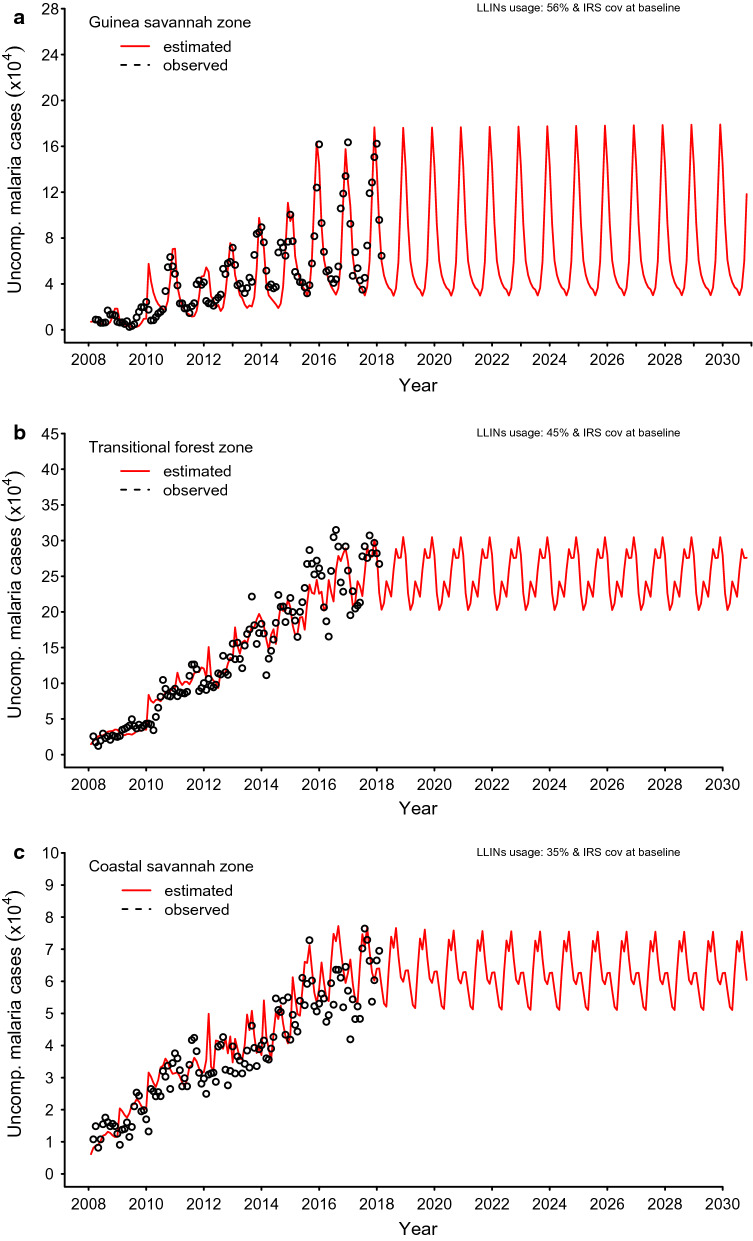
Model run time is 1988 to 2030. Steady state period spans from 1988 to 1997, 1998 to 2017 previous interventions implemented and reporting rates on DHIMS introduced. Data fitting and calibration from 2008 to 2017 for the **a** Guinea savannah, **b** transitional forest and **c** coastal savannah

**Fig. 5:**
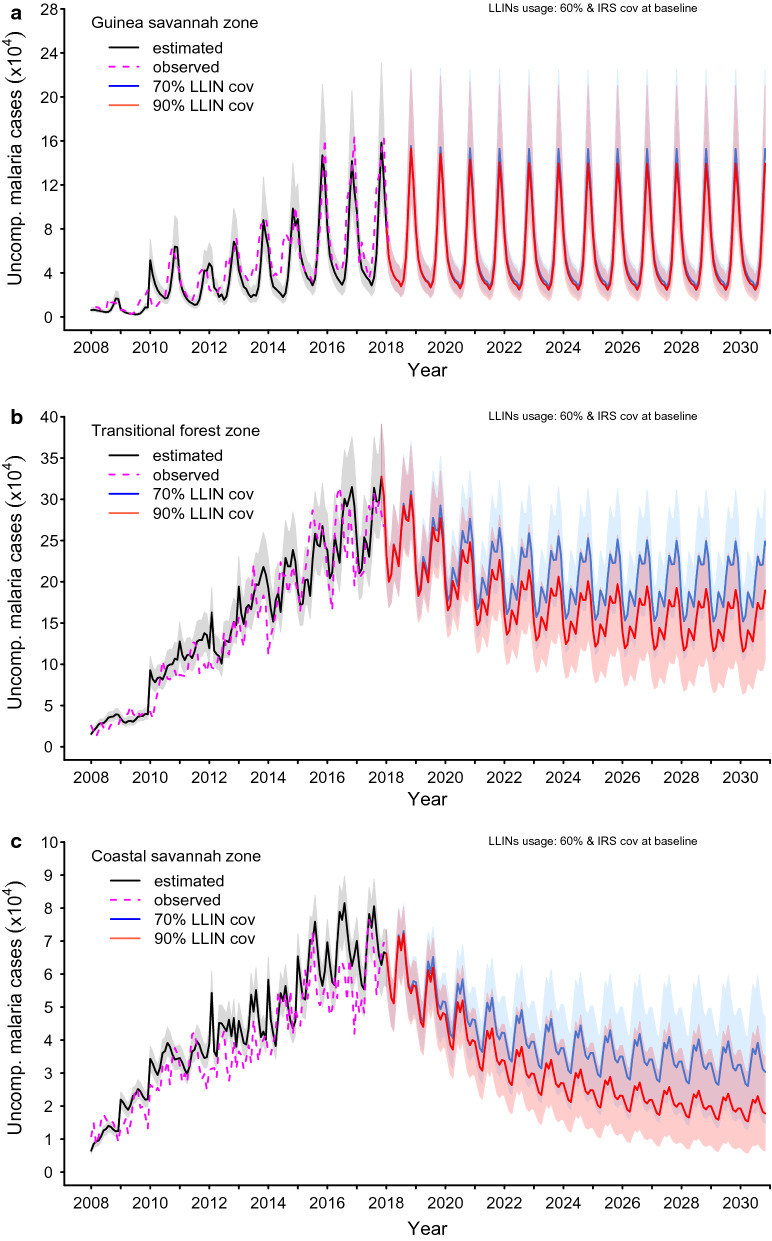
Impact of attaining various levels of LLINs coverage within a 3-year implementation programme at a usage level of 60.0% while maintaining IRS coverage and PE at prevailing baseline levels in the **a** Guinea savannah, **b** transitional forest and **c** coastal savannah

**Fig. 6:**
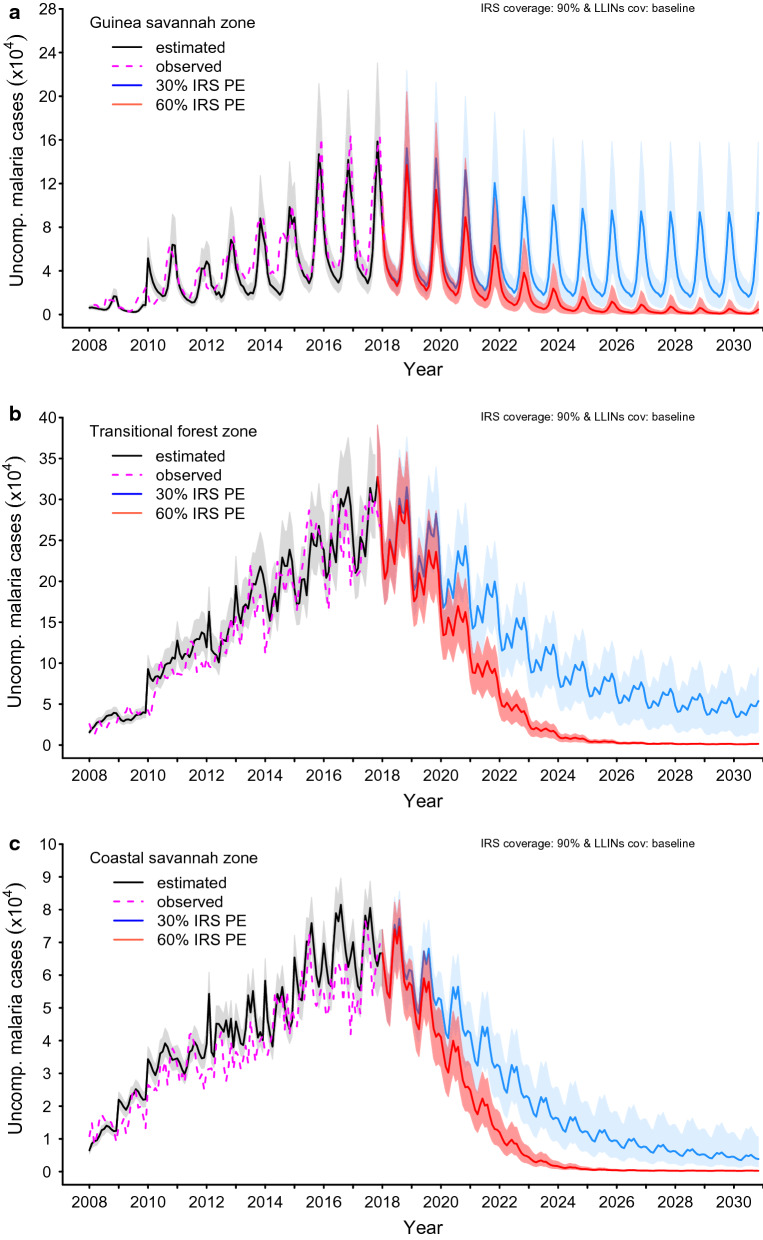
Impact of attaining various levels of IRS coverage within a 5-year implementation programme at various protective efficacy (PE) while maintaining IRS coverage at 90.0% and PE, coverage levels and usage of LLINs at prevailing baseline levels in the **a** Guinea savannah, **b** transitional forest and **c** coastal savannah

As shown in Figs. 4a–c, the parameters were calibrated with data from 2008 to 2017, as shown in Additional file: S1 Figs. 4, 5 and 6. These figures depict the baseline scenarios for all zones.

Figure 4a shows that the incidence of uncomplicated malaria in the Guinea savannah follows the seasonal rainfall patterns, which is generally of a single peak. While similar patterns are observed in the transitional forest and coastal savannah, incidence of uncomplicated malaria peaks twice a year. As depicted in Fig. 4b, c below, there is, however, a relatively less prominent second season in the coastal savannah compared to that of the transitional forest zone.

Estimated burden of all clinical cases of malaria (uncomplicated and severe) in the baseline year of 2018 was 219 (95% p.CI [153, 315])/1000 population, 261 (95% p.CI [220, 312])/1000 population and 139 (95% p.CI [117, 154])/1000 population for the Guinea savannah, transitional forest, and coastal savannah zones respectively. However, reported cases of uncomplicated malaria only in 2018 at the health facilities were estimated to be 173 (95% p.CI [121, 250])/1000, 199 (95% p.CI [168, 238])/1000 and 104 (95% p.CI [88, 115])/1000 population in the Guinea savannah, transitional forest, and coastal savannah zones, respectively.

### Predictions

Results of scaled up interventions implemented for 3 years to achieve universal coverage levels with respect to LLINs, and 5 years to achieve targeted coverage levels of IRS, in the three zones, were simulated from 2018 to 2030 under various intervention scenarios as presented in the sections below.

### Impact of LLIN interventions

#### LLIN coverage of 70.0% and 90.0% at baseline usage (56.0%, 45.0% and 35.0% for Guinea savannah, transitional forest and coastal savannah, respectively)

The impact of increasing the universal coverage levels of ITNs/LLINs was tested with selected scenarios for the various zones. Results obtained from the models after simulation show that achieving elevated levels of LLIN coverage of 70.0% and 90.0%, given usage at the baseline level of protective efficacy of LLINs at 40.0% and IRS at 30.0% [31], while keeping the coverage levels of IRS at baseline at 2018, leads to a 2.5% and 8.9% reduction in uncomplicated cases in the Guinea savannah, 8.2 and 17.3% in the transitional forest and 9.9% and 19.8% in the coastal savannah, respectively, Additional file 2: S2 Fig. 1.

For predictions of all reported clinical incidence of malaria (uncomplicated and severe), the corresponding reductions in the incidence rates for all the zones are shown in Table 2.

**Table 2:** Predictions of reported clinical malaria (uncomplicated and severe cases) incidence rate per 1000 population with 95% pseudo-confidence intervals (95% p.CI) for various coverage levels of LLINs and IRS and LLIN usage (%) or IRS protective efficacy (PE) (%) at 2020 and by 2030 by zone

Zone	Intervention	Coverage (%)		Usage (%)	PE (%)		Incidence rate/1000 population	
							(95% p.CI) by year^a^	
		LLIN	IRS^b^	LLIN	LLIN	IRS	2020	2030
Guinea savannah	LLINs	70	17	56	40	30	169 (117, 245)	168 (116, 245)
				60	40	30	166 (114, 242)	165 (112,241)
				80	40	30	150 (97, 223)	148 (91,222)
		90	17	56	40	30	160 (108, 245)	155 (100, 230)
				60	40	30	156 (104, 230)	151 (94, 225)
				80	40	30	136 (84, 206)	125 (62, 196)
Transitional forest	LLINs	70	0	45	40	30	189 (157, 226)	177 (139, 215)
				60	40	30	171 (139,206)	148 (103, 186)
				80	40	30	146 (115,179)	107(57,145)
		90	0	45	40	30	179 (148, 226)	159 (109, 190)
				60	40	30	158 (126, 191)	113 (64, 151)
				80	40	30	130 (100, 160)	60 (22, 93)
Coastal savannah	LLINs	70	0	35	40	30	97 (79, 110)	87 (63, 104)
				60	40	30	77 (60, 91)	51 (26,78)
				80	40	30	62 (47, 77)	27 (10,55)
		90	0	35	40	30	92 (74, 110)	73 (47, 94)
				60	40	30	69 (53, 83)	31 (12, 58)
				80	40	30	53 (39, 67)	11 (4, 28)

^a^95% p.CI 2.5 and 97.5% quantiles around the mean of the distribution of the predicted clinical cases of malaria

^b^Baseline IRS coverage

### LLIN coverage of 70.0% and 90.0% and usage at 60.0% across zones

When coverage levels were maintained at 70.0% and 90.0%, in all zones, reductions in predicted uncomplicated cases of 4.2% and 11.3%, respectively, in the Guinea savannah, 20.0% and 32.8% in the transitional forest, and 36.9% and 51.3% in the coastal savannah, were observed with an increased level of usage of LLINs of 60.0%. PE of LLINs and IRS remained at baseline levels; see Additional file 2: S2 Fig. 1 and Fig. 5.

The incidence rates corresponding with an increased LLIN usage of 60.0% in the Guinea savannah were 166 (95% p.CI [114, 242])/1000 and 156 (95% p.CI [104, 230])/1000 population in 2020, and 165 (95% p.CI [112, 241])/1000 and 151 (95% p.CI [94, 225])/1000 population by 2030, respectively, for LLIN coverage levels of 70.0% and 90.0%, as shown in Table 2 and Fig. 5.

The rates predicted in the transitional forest and the coastal savannah for elevated use of LLIN at 60.0%, and for LLIN coverage levels of 70.0% and 90.0% by 2020 and 2030, are also shown in Table 2 and Fig. 5.

### LLIN coverage of 70.0% and 90.0% and usage of 80.0% across all zones

A further proportion of predicted cases of reported uncomplicated malaria can be averted when the LLINs usage level is increased to 80.0%. The proportions of predicted cases averted in the Guinea savannah, transitional forest, and coastal savannah are 13.5%, 36.6%, and 56.7% for a 70.0% LLIN coverage and 24.4%, 53.2% and 69.0%, for LLIN coverage of 90.0%, respectively, across all the zones (Additional file 2: S2 Fig. 1).

At 80.0% usage level of LLINs, the rates for various zones are shown in Table 2. They demonstrate considerable reductions in incidence of malaria in these zones.

### Impact of IRS interventions

#### IRS coverage of 90.0% and PE of 30.0% and 60.0%, LLIN coverage and usage at baseline levels (66.0% and 56.0% in the Guinea savannah, 51.0% and 45.0% in the transitional forest and 50.0% and 35.0% in the coastal savannah, respectively)

A relatively higher number of cases of uncomplicated malaria could potentially be averted with a 90.0% IRS coverage level and PE levels of 30.0% and 60.0% across all the zones (Fig. 6 and Additional file 2: S2 Fig. 2).

In the Guinea savannah, averting 72.0% and 79.0% of uncomplicated cases could be attained by 2030 for IRS PE at 30.0% and 60.0% levels, respectively (Additional file 2: S2 Fig. 2 and Fig. 6).

The impact of these declines in the Guinea savannah on the incidence of all cases of malaria cases was observed to be 146 (95% p.CI [95, 218])/1000 and 105 (95% p.CI [59, 164])/1000 population by 2020, and 102 (95% p.CI [36, 169])/1000 and 6 (95% p.CI [1, 15])/1000 population by 2030, for a 30.0% and 60.0% PE respectively, for an IRS coverage of 90.0% (Table 3).

**Table 3:** Predictions of reported clinical malaria (uncomplicated and severe cases) incidence rate per 1000 population with 95% pseudo-confidence intervals (95% p.CI) for various coverage levels of LLINs and IRS and LLIN usage (%) or IRS protective efficacy (PE) (%) in 2020 and by 2030 by zone

Zone	Intervention	Coverage (%)		Usage (%)	PE (%)		Incidence rate/1000 population	
							(95% p.CI) by year^a^	
		LLIN	IRS	LLIN	LLIN	IRS	2020	2030
Guinea savannah	IRS	66	90	56	40	30	146 (95, 218)	102 (36, 169)
				56	40	60	105 (59, 164)	6 (1, 15)
				56	40	80	78 (39, 125)	0 (0, 1)
Transitional forest	IRS	51	90	45	40	30	159 (128, 192)	35 (12,59)
				45	40	60	121 (94, 149)	1 (1,1)
				45	40	80	99 (75, 122)	0 (0, 0)
Coastal savannah	IRS	50	90	35	40	30	75 (59, 89)	8 (3, 20)
				35	40	60	53 (40, 65)	0 (0, 0)
				35	40	80	40 (30, 51)	0 (0, 0)

^a^95% p.CI 2.5 and 97.5% quantiles around the mean of the distribution of the predicted clinical cases of malaria

Likewise, in the transitional forest zone, potentially 75.7% of uncomplicated malaria cases could be averted with an IRS coverage of 90.0% and PE of 30.0%, and 78.5% for an IRS PE of 60.0%, by 2030 (Additional file 2: S2 Figs. 2 and Fig. 6).

Correspondingly, the rates of incidence of all cases of malaria were 159 (95% p.CI [128, 192]) and 121 (95% p.CI [94, 149]) for an IRS PE of 30.0% and 60.0%, respectively, by 2020, and 35 (95% p.CI [12, 32]) and 1 (95% p.CI [1]) for an IRS PE of 30.0% and 60.0%, respectively, by 2030 (Table 3 and Fig. 6).

For IRS only, uncomplicated cases averted, as shown in Fig. 6 and Additional file 2: S2 Fig. 2, was 78.5% versus 80.9% for a 90.0% IRS coverage with 30.0% and 60.0% levels of PE, respectively, by 2030. The corresponding incidence rates for all cases of malaria following the attainment of these intervention targets by 2020 and 2030, respectively, are shown in Table 3 and Fig. 6.

### Impact of deploying LLINs and IRS

#### LLIN coverage at 80.0% and IRS coverage at 80.0% with LLIN usage and IRS PE at baseline settings (56.0% and 30.0% in the Guinea savannah, 45.0% and 30.0% in the transitional forest and 35.0% and 30.0% in the coastal savannah, respectively)

Achieving 80.0% LLIN and IRS coverage while maintaining LLIN usage and IRS PE at baseline potentially results in 30.8%, 58.0% and 64.7% of reported uncomplicated malaria cases averted in the Guinea savannah, transitional forest, and coastal savannah, respectively (Additional file 2: S2 Fig. 3).

**Fig. 7:**
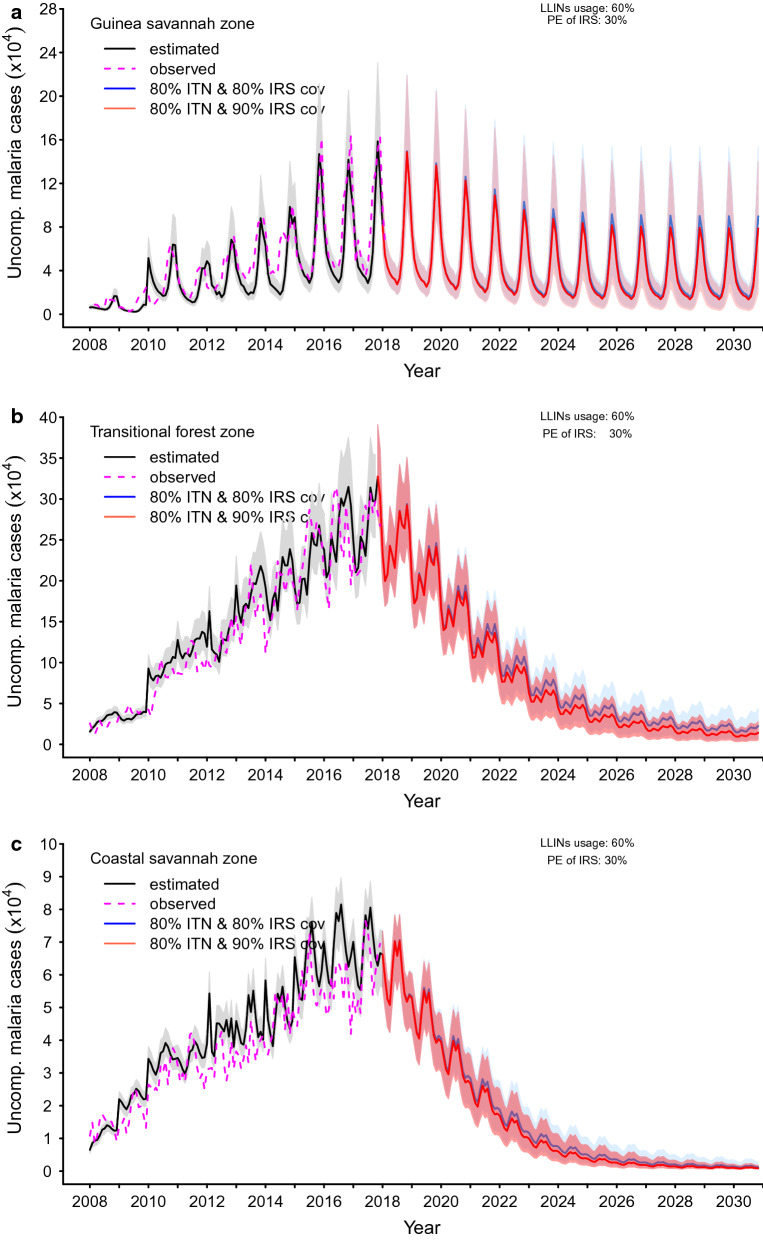
Impact of attaining a combination of various levels of LLINs and IRS coverage within 3 and 5 year implementation programme respectively at baseline protective Efficacy (PE) of IRS (30.0%) and elevated level of LLINs (60.0%) usage in the **a** Guinea savannah, **b** transitional forest and **c** coastal savannah

The proportions of malaria cases averted for implementing an 80.0% LLIN and IRS coverage at baseline LLIN usage and IRS PE was likely to give rise to reductions in incidence, as shown in Table 4 and Fig. 7. When the coverage levels of LLIN and IRS were both increased to 90.0%, but all other scenarios remained as in the previous scenario, cases averted were observed to be 39.1%, 64.1% and 69.0% in the Guinea savannah, transitional forest and coastal savannah zones, respectively, as shown in Additional file 2: S2 Fig. 3. The corresponding rates for the various zones are captured in Table 4 and Fig. 7.

**Table 4:** Predictions of reported clinical malaria (uncomplicated and severe cases) incidence rate per 1000 population with 95% pseudo-confidence intervals (95% p.CI) for various coverage levels of LLINs and IRS and LLIN usage (%) or IRS protective efficacy (PE) (%) in 2020 and by 2030 by zone

Zone	Intervention	Coverage (%)		Usage (%)	PE (%)		Incidence rate/1000 population	
							(95% p.CI) by year^a^	
		LLIN	IRS	LLIN	LLIN	IRS	2020	2030
Guinea savannah	LLIN and IRS	80	80	56	40	30	144 (93, 214)	103 (37, 170)
		90	90	56	40	30	136 (86, 204)	83 (20, 146)
		80	80	60	40	30	140 (89, 210)	98 (33, 165)
		80	90	60	40	30	137 (86, 206)	86 (23,151)
Transitional forest	LLIN and IRS	80	80	45	40	30	150 (120, 183)	29 (9, 51)
		90	90	45	40	30	142 (113, 173)	16 (5,29)
		80	80	60	40	30	133 (103, 163)	16 (5, 30)
		80	90	60	40	30	129 (100, 159)	10 (4, 20)
Coastal savannah	LLIN and IRS	80	80	35	40	30	72 (56, 85)	7 (3, 18)
		90	90	35	40	30	67 (52, 80)	4 (2, 10)
		80	80	60	40	30	55 (41, 68)	2 (1,6)
		80	90	60	40	30	53 (39, 66)	2 (1, 4)

^a^95% p.CI 2.5 and 97.5% quantiles around the mean of the distribution of the predicted clinical cases of malaria

#### LLIN coverage at 80.0% and IRS coverage at 80.0% with LLIN usage at 60.0% and IRS PE at baseline settings (30.0% in the Guinea savannah, transitional forest, and coastal savannah)

Given coverage levels of LLIN and IRS of 80.0%, but with LLIN usage increased to 60.0% in all zones, 33.0%, 65.8% and 74.6% of uncomplicated cases of malaria could be averted in the Guinea savannah, transitional forest, and coastal savannah, respectively (Additional file 2: S2 Fig. 3). Various rates corresponding to these reductions for all cases of malaria by 2020 and 2030, respectively, are as shown in Table 4 and Fig. 7.

#### LLIN coverage at 80.0% and IRS coverage at 90.0% with LLIN usage of 60.0% and IRS PE at baseline settings (30.0% in the Guinea savannah, transitional forest, and coastal savannah, respectively)

The corresponding proportions of cases potentially averted, with LLIN coverage of 80.0% and usage of 60.0%, deployed in combination with an IRS coverage of 90.0%, as shown in Additional file 2: S2 Fig. 3, could be 37.7% for uncomplicated malaria in the Guinea savannah. The associated reductions in the incidence of all clinical cases of malaria by 2020 and 2030, respectively, are depicted in Table 4 and Fig. 7.

In the transitional forest zone, 68.3% of uncomplicated cases were predicted to be averted by 2030 (Additional file 2: S2 Fig. 3). The associated incidence rates, as shown in Table 4, were 129 (95% p.CI [100, 159])/1000 and 10 (95% p.CI [4, 20])/1000 population for the transitional forest by the years 2020 and 2030, respectively.

Similarly, for the coastal savannah, the proportion of uncomplicated malaria cases potentially averted was 76.1% (Additional file 2: S2 Fig. 3). Correspondingly, incidence rates for all clinical cases of malaria under this scenario were predicted to be 53 (95% p.CI [33, 34])/1000 and 2 (95% p.CI [1, 4])/1000 population by 2020 and 2030, respectively (Table 4 and Fig. 7).

## Discussion

The potential impact of malaria interventions was investigated by simulating various implementation scenarios, while taking into account the diversity of morbidity in the three ecological zones across Ghana. These investigations, which were conducted spanning 2018 to 2030, also assessed the prospects of achieving some goals of the Ghana National Malaria Strategic Plan, 2014–2020, as well as those of the World Health Organization (WHO) Global Technical Strategy milestones on malaria control [1].

The models take into account the population sizes of the different transmission settings. Differences in transmission potential for young children, adults, and pregnant women were also considered. The gradual improvement in the data capture and reporting, through the DHIMS infrastructure, at the district level in government health facilities and faith-based private facilities across the country, was accounted for by allowing for various levels of reporting and system improvements from 2008 to 2018. Years of improvement in all suspected cases receiving a malaria diagnostic test was also incorporated (Fig. 3) [32, 35].

The roll out of LLINs on a large scale basis in Ghana began in 2003 [32]. This resulted in a substantial improvement in the proportion of households with at least one LLIN, as well as at least one LLIN per every two members of a household (universal coverage) across the country [24]. For instance, as at 2016, the average proportions of households with at least one LLIN were 89.0%, 74.8%, and 70.0%, compared to 59.0%, 42.5% and 37.6% in 2008 for the Guinea savannah, transitional forest, and coastal savannah zones, respectively [21, 24]. On the other hand, the average coverage levels (universal) of LLINs in 2016 were 65.7%, 50.5% and 49.9% for the Guinea savannah, transitional forest, and coastal savannah zones, respectively [24]. These achievements have largely contributed to the gradual decline in the prevalence of malaria among children aged 6–59 months of age, with the latest (2016) measurement's having fallen to 21.0%, from 27.0% in 2014 [24].

ITN/LLIN usage is relatively low across the country. On average, 56.0%, 45.0%, and 35.2% of the populations in the Guinea savannah, transitional forest and coastal savannah zones, respectively, were reported to have slept in an ITN/LLIN in 2016, a marginal increase from 47.1%, 45.6%, and 32.5% in 2008, for children under the age of five years [21, 24, 36]. These observations follow the results of this study, which suggest that ITN or LLIN usage could be low given the current level of coverage and incidence of malaria across all of the zones. The results from the models show that, with elevated levels of usage of LLINs, which improves PE, a significant number of predicted incidence cases could be averted.

For example, as described earlier, the predicted cases averted by increasing the coverage levels of LLINs to targeted levels of 70.0% and 90.0%, during a three year implementation campaign period, leads to only a marginal improvement from the baseline scenario, without a corresponding increase in the PE of the LLINs (Additional file 2: S2 Fig. 1). This observation may explain why the relatively high universal coverage levels of LLINs currently observed (at least 50.0% across all zones as at 2016) may not be reducing the level of predicted cases as expected.

Even though LLIN deployment has been reported to be one of the most efficient packages, which can lead to a 75.0% reduction in disease, in much of Africa [37], averting more predicted cases through LLINs may only be possible through intensifying the campaign to persuade the population to comply with proper LLIN usage, while continuous efforts are made to sustain the coverage already achieved. Many reasons have been reported for people’s not sleeping in ITN/LLIN including an inability to hang them, real or perceived health concerns, difficulty in breathing when sleeping under them, and other factors [38–40].

This calls for further and continuous advocacy on the usage of ITNs/LLINs, including the use of formal education channels, and community hang-up/social behaviour communication change campaigns, on the proper usage of the LLINs, while highlighting the potential biting patterns of mosquitoes to avert unnecessary outdoor exposure [18, 19].

Given the proven efficacy of LLINs, and the relatively high coverage levels currently prevailing in the various zones, correspondingly higher reductions in the burden of malaria could have been achieved if the usage of these LLINs was equally as high, as demonstrated throughout the results of various intervention scenarios simulated in this study with increasing levels of usage (Additional file 2: S2 Fig. 1).

Following the WHO guidelines for vector control, Ghana may have attained a high enough LLIN coverage level in selected areas, especially in the Guinea savannah zone, where transmission is highly seasonal and coverage is relatively higher, to begin the roll out of IRS on a targeted large-scale basis as a complementary vector control measure [8, 40].

However, relative to LLINs, the coverage of IRS is by far the lowest across the country. Although parts of the Guinea savannah and the Transitional forest zones have had some implementation of IRS on pilot bases, studies of any such activities rolled out in the Coastal savannah are yet to be cited [32, 34, 41].

It was shown in parts of the Guinea savannah that districts where IRS was deployed compared to non-IRS districts resulted in a reduction of 39.0%, on average, in malaria incidence during the six months after spraying. These gains were, however, reversed when the IRS activities were not sustained [41, 42].

Results in this study also show that a potential increase in the reduction of predicted cases of malaria, from 48.9% to 90.4%, could be attained with an increased deployment of IRS in the various zones, for varying levels of PE of a spraying programme that takes up to five years to attain and maintain these coverage levels (80.0% and 90.0%), Additional file 2: S2 Fig. 2. At these levels of decline, pre-elimination could be in sight, as observed in the incidence rates of 1 (95% p.CI [1])/1000 population or less. This is possible if a 90.0% coverage of IRS is attained within five years and maintained up to 2030 across the country (Table 3).

IRS might hold a promise of averting more cases of malaria compared to LLINs, given the relatively low level of dependence on human behaviour for usage. However, the feasibility of rolling out IRS as an additional intervention to LLNs on a large or targeted basis may depend on the level of community acceptability, and the considerable additional cost given the limited operational budget space.

As shown in Fig. 7 and Table 4, LLIN usage in the presence of targeted IRS deployment seems to avert a substantial number of incidence cases in all zones. This reinforces the importance of using the LLIN as recommended, in order for the possible optimal benefit of malaria prevention to be realized.

Evidence from some previous field and modelling studies suggests that combining the LLINs and IRS offers higher protective effectiveness. For instance, the impact of the combination compared to only IRS was found to be OR = 0.71 (95% CI (0.59–0.86)) in Equatorial Guinea and OR = 0.63 (95% CI (0.50–0.79)) in Mozambique. Another study in Kenya reported similar results with PE of ITN and IRS, compared with ITN only, to be 62% (95% CI ( 0.50–0.72)) [43, 44]. Similarly, a cluster randomized study in the north-west of Tanzania showed that there was an enhanced benefit of combined ITN and IRS utilization. The odds of infection for a population that used ITNs in village clusters which were sprayed was reported to be considerably (two-thirds) lower than those with either ITN or IRS (OR = 0.34, 95% CI 0.23–0.53). This reduction was significantly higher compared to using ITN only (OR = 0.83), and yet greater still than reported for village clusters sprayed with IRS (OR = 0.41) only [45]. These findings are largely consistent with those reported in this study in Additional file 2: S2 Figs. 1, 2 and 3. Therefore, combining both LLINs and IRS will likely contribute very significantly, not only to averting many more predicted cases across Ghana, but to driving the annual incidence of malaria presented at the health facilities down towards pre-elimination levels if IRS coverage is scaled up across all three zones, and LLIN usage is improved substantially, a combination which has been suggested to be justified [44, 46, 47].

All investigations in this study considered hypothetical scenarios of deploying both LLINs and IRS. Moreover, IRS was considered as a supplementary intervention to LLIN. For practical and financial considerations, it may be infeasible to achieve universal coverage of both LLINs and IRS across the country. This makes efforts towards improving the effectiveness of LLIN, at the already high coverage levels, imperative; otherwise, it amounts to not achieving value for money for the investment over the years.

Therefore, as continuous efforts are being made by the NMCP and other stakeholders to scale up various vector control measures across the country, an even stronger advocacy needs to be made for education of the population through various channels such as radio, television messages and programmes, and community durbars, on the uptake of the various malaria interventions, especially LLINs [48, 49].

There are possible high levels of LLIN non-usage in Ghana, at 58.0% (2016), which is relatively higher compared to its neighbours, Benin at 28.9% (2017), Burkina Faso at 33.0% (2014) and Côte d’Ivoire at 49.6% (2016). As such, the community health officers stationed in the various Community-Based Health Planning Services (CHPS) zones may be of great use in undertaking these additional tasks of educating and mounting hang-up campaigns and other means of communication to improve the usage of LLINs [36, 38, 50, 51].

From the results thus far, it is unlikely, with the current observed rate of decline, that Ghana will achieve the principal target of reducing the burden of malaria by 75.0% (which translates to 47 cases per 1000 population per year, using cases reported in 2012 as baseline) by close of 2020, as projected in the National Malaria Strategic plan of 2014–2020, even though large declines have been achieved with malaria-attributable deaths [4]. Meeting the goals of the strategic plan by 2030 may require a full scale deployment of IRS in targeted districts and communities complementary to LLINs in all the zones to at least 80.0% coverage, using insecticides with high level of protective efficacy (Table 4).

The relatively high treatment-seeking (72.0%) and diagnosis (90.0%) levels for the Guinea savannah, transitional forest, and coastal savannah were taken into account when testing the impact of the various interventions. Attaining improved coverage levels of vector control interventions across the country will require more investment in a multi-pronged approach to roll out interventions such as LLINs and IRS (in targeted districts) to prevent cases and to treat cases concurrently. This should occur along with rallying all citizenry to improve usage of LLINs, to seek treatment promptly, and to invest in personal protection.

## Conclusions

This study has shown that it is possible to achieve targets set out by the NMCP, and those of the Global strategy for malaria, using current interventions, if compliance to their recommended applications is improved. Therefore, any programmes and strategies which would further increase the patronage and proper and continuous use of ITN/LLIN should be encouraged and supported. As shown in the results, improvement in the coverage of LLIN only, without a corresponding improvement in usage, does not reduce the incidence of malaria in the population.

With respect to IRS, districts with incidence rates of malaria above zonal average levels could be targeted for IRS to complement LLINs, as recommended by the WHO, since the LLIN coverage is relatively high. If desired levels of malaria-related morbidity are to be attained, as projected by the National strategic policy of 2014–2020 [4], then a rapid and momentous effort needs to be made to improve upon the uptake and sustained usage of the LLINs, while consideration is given to targeted IRS, especially in high risk districts in the transitional forest and coastal savannah zones.

The findings of this study may contribute to future policy formulation for malaria control in the country.

## Supplementary information

**Additional file 1:** (DOCX 164 KB).

**Additional file 2:** (DOCX 1595 KB).

## Data Availability

The authors do not have the rights to share the temperature and rainfall data which can obtained from client@meteo.gov.gh. The health facility based malaria data can be requested at nmcp@ghsmail.org.

## Abbreviations

NMCP: National Malaria Control Programme
ITN: Insecticide-treated bed nets
LLIN: Long-lasting insecticide-treated bed nets
IRS: Indoor residual spraying
USAID: United States Agency for International Development
PMI: President’s Malaria Initiative
AIDS: Acquired immune deficiency syndrome
ACTs: Artemisinin-based combination therapy
IPTp: Intermittent preventive treatment of malaria in pregnancy
SP: Sulfadoxine-pyrimethamine
SIRS: Susceptible infected recovered susceptible
BR: Biting rate
HLC: Human landing catches
Rf: Rainfall
Kv: Environmental carrying capacity
DHIMS: District Health Information Management System
DHS: Demographic and Health Survey
MICS: Multiple Indicator Cluster Survey
SMC: Seasonal malaria chemotherapy
ABC: Approximate Bayesian computation
PE: Protective efficacy
MSAT: Mass screen and treat
WHO: World Health Organization
IPTi: Intermittent preventive treatment of malaria in infants
SACEMA: South African Centre for Epidemiological Modelling and Analysis
DST-NRF: Department of Science and Technology-National Research Foundation
ICTS: Information and Communication Technology Services
GetFUND: Ghana Education Trust Fund
CHPS: Community-Based Health Planning Services
