# Supplementary material for: Accounting for regional transmission variability and the impact of malaria control interventions in Ghana: a population level mathematical modelling approach
Source: Malar J. 2020 Nov 23;19:423. doi: 10.1186/s12936-020-03496-y (PMC7684904; doi:10.1186/s12936-020-03496-y)
Supplement: Supplementary file 1 — Additional file 1: (DOCX 164 KB). [file 12936_2020_3496_MOESM1_ESM.docx]

**S2 Fig 1: Impact of attaining various levels of LLINs coverage and usage while maintaining IRS coverage and PE at prevailing baseline levels on predicted reported uncomplicated malaria cases across zones**. **BaseUSE is LLIN usage at baseline (56.0%, 45.0% and 35.0% in the Guinea savannah, Transitional forest and Coastal savannah respectively) by 2030.**

**S2 Fig 2: Impact of attaining various levels of IRS coverage and Protective Efficacy (PE) while maintaining LLIN coverage and PE at prevailing baseline levels on predicted reported uncomplicated malaria cases across zones by 2030.**

**S2 Fig 3: Impact of attaining various levels of LLINs and IRS coverages, LLIN usage and IRS Protective Efficacies (PE) on predicted reported uncomplicated malaria cases across zones. BaseUSE is LLIN usage at baseline (56.0%, 45.0% and 35.0% in the Guinea savannah, Transitional forest and Coastal savannah respectively) by 2030.**
