# Supplementary material for: Accounting for regional transmission variability and the impact of malaria control interventions in Ghana: a population level mathematical modelling approach
Source: Malar J. 2020 Nov 23;19:423. doi: 10.1186/s12936-020-03496-y (PMC7684904; doi:10.1186/s12936-020-03496-y)
Supplement: Supplementary file 2 — Additional file 2: (DOCX 1595 KB). [file 12936_2020_3496_MOESM2_ESM.docx]

# Accounting for regional transmission variability and the impact of malaria control interventions in Ghana: A population level mathematical approach.

**Supplementary file**

**Introduction**

Malaria infection is still a major public health concern in Ghana and the entire population is at a high risk of infection all year round. Younger children are more at risk of clinical expression of the disease compared with adults due to acquired immunity over time following repeated exposure to parasite infection [1].

As described elsewhere, transmission dynamics vary considerably across the country following the dynamics of the ecology in three geographical zones; namely the Guinea savannah, Transitional forest and the Coastal savannah [2].

The characteristics of these different ecological settings have been shown to be principal factors in the transmission dynamics of malaria morbidity in the various zones. In evaluating the impact of various interventions on the transmission of malaria, it is therefore appropriate to consider dynamics of the environment in the respective zones [2].

**Transmission model**

A model was developed and replicated for the three zones. The model is a human-vector coupled with the vector component dependent on the environmental variables such as monthly average temperature (°C) and rainfall (mm). It was also structured to reflect in some parts the health system architecture, especially with respect to malaria morbidity and mortality in Ghana. The diagram in Fig 1 shows the structure of the model.

**Demographics**

Though the model is not age structured, the recruited population is divided mainly into three, one for children under 6 years (**Sn**), who are relatively naïve, children born with a congenital infection (2.5%), and the third for all 6 years and above (**Snn**).

**Base model diagram**

**Fig 1 Malaria transmission model showing various compartments of both human and vector populations.**

**Human population: S (Sn**, **Snn** and **Snnp** representing the susceptible human compartments respectively for new births and children under 6 years of age, adults and pregnant women) represents the susceptible class with the **Snnp** being the susceptible pregnant women drawn from the adult population, **Sn**. **L (L1**,**L2** and **L3** respectively for new births and children under 6 years of age, adults and pregnant women**)** represent the latent infection period before the onset of gametocytes. **Ic**, **Ia**, **Is** and **Ism** compartments represents symptomatic infection (clinical infection), asymptomatic infection, severe infection and sub-microscopic infection respectively. Pregnant women attend antenatal clinic (ANC) without an infection, **IANCN** or progress from **L3** into **IANCP** once infected.**Tr1**, **Tr2** and **Tr3** represent the treatment sought for confirmed uncomplicated malaria (**Ia**), severe malaria (**Is**) and routine monthly SP prophylaxis for pregnant women at ANC, **Trf1**, **Trf2** and **Trf3** represent respective treatment failure due to adherence and possible drug resistance for the three latter treatment options. **Vector population:** **Lv** represents larva population and **Sm** susceptible mosquitoes. Exposed mosquitoes are captured in **Em** compartment. Whereas infectious mosquitoes are in the **Im** compartment. FOI is force of infection.

This is to account for the different risk profile of infection for children under 6, pregnant women and others not in these categories [3,4]. **Sn**, **Snn** and **Snnp** therefore are compartments for infants and young children (less than 6 years old), all others 6 years and above and susceptible pregnant women respectively, Fig 1, and 1a.

To account for malaria in pregnancy, a compartment for pregnant women in the population (**Snnp**) was added to allow for the dynamics of infection and Antenatal Clinic (ANC) attendance since these cases of malaria are captured at the ANC. It is estimated that, pregnant women make up 2.5% of the population and these are drawn from the **Snn** compartment as shown on Fig 1a [5].


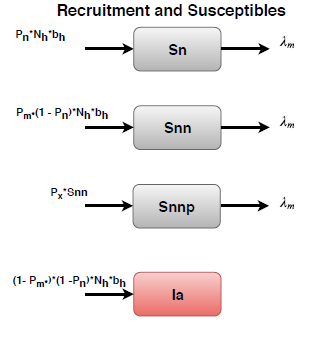


**Fig 1a Model compartments for recruitment and susceptible populations**

Congenital infection with malaria is an important component of the burden of malaria among neonates. In Ghana, studies have shown that, 2.2% or more of neonates are infected with malaria at birth even though the recommended Intermittent Preventive Treatment in pregnancy (IPTp) intake among pregnant women across the country is more than a third. Though at these level of prevalence it may seem insignificant, a proportion of the new borns with a congenital infection are recruited into the asymptomatically infected (**Ia)** compartment [3,4,6,7].

From Fig 1a, **P_n_** _,_ **P_m_** and **P_x_** are respectively proportions of the children under, population 6 and above and the proportion of pregnant women from the adult population that are recruited into the susceptible compartments. **N_h_** and **b_n_** represent the population size and life expectancy. $\boldsymbol{\lambda}_{\boldsymbol{m}}$ represents the force of infection from mosquito to human transmission.

**Exposed/Latent period in humans**

Upon infection (through force of infection $\boldsymbol{\lambda}_{\boldsymbol{m}}$ described later), following a successful mosquito bite on a susceptible human, progress is made into the latent state (liver stage infection) of infection (**L1, L2** and **L3** respectively for the children under 6 years, the rest of the population and pregnant women ) as shown on Fig 1b.


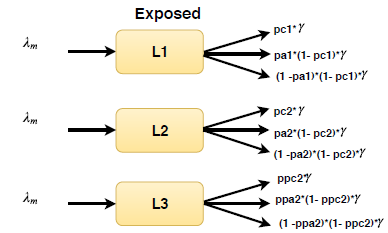


**Fig 1b Model compartments for exposed populations**

Until the blood stage **Ic** (clinical malaria infection), **Isd** (severe disease),**Ia** (asymptomatic infection) and potentially **Ism** (sub-microscopic infection) it is assumed that all latent states (**L1**, **L2** and **L3**) infections are non-infectious and undetectable by either Rapid Diagnostic Test (RDT) or microscopy. **pc1**, **pc2** and ppc are the probabilities of children under 6, adults above 6 years and pregnant women respectively developing symptomatic malaria after being exposed whiles **pa1**, **pa2** and **ppa** are respectively for developing an asymptomatic malaria. Based on the conditional probability applied, the rest of the exposed develop sub-microscopic infection with a probability less those of symptomatic and asymptomatic malaria as shown on Fig 1 and 1b. $\boldsymbol{\gamma}$ is the duration of incubation before the blood stages of infection occurs.

**Infected/Infectious stages**

While the asymptomatically infected population in **Ia or Ism** could make a transition into symptomatically infected (**Ic)** compartment, perhaps due to waning immunity or being infected again (superinfection in Fig 1c, **SI** in Fig 1)), a proportion of those in the **Ic** compartment could also recover naturally into **Ia** from where, if parasites loads are very low may not be detectable and therefore classified into the sub-microscopic infection (**Ism)** state.


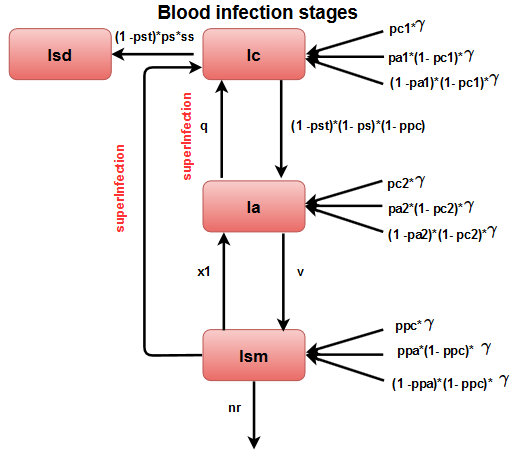


**Fig 1c Model compartments for infectious (symptomatic, asymptomatic and sub-microscopic) populations**

From the **Ism** stage, they could recover from their infection naturally at rate **nr** to become susceptible **Snn** with time.

A proportion of the sub-microscopically infected population **Ism**, could progress into the **Ia** state or **Ic** upon being infected again subsequent to the pre-existing infection (superinfection) or having their immunity waning and therefore giving way to an increase in parasite load. Likewise, a proportion of the population with severe malaria infection (**Isd**) may die without or delayed treatment however the compartment for malaria related mortality is not shown.

Given the reported health seeking behaviour (**pst**) of the infected population, the likelihood of receiving a diagnostic test at the health facility (**pt1**) and the sensitivity of the various diagnostic tests kits (**RDTMicSens**) in various zones, the symptomatically infected are likely to be diagnosed correctly from the **Ic** and **Isd** states and treated appropriately in **Tr1** and **Tr2** respectively for an uncomplicated malaria or a severe malaria according to national treatment guidelines.

**Treatment adherence and Drug resistance**

Ghana currently uses three Artesunate combination therapies (ACTs) (namely: Artesunate Amodiaquine (ASAQ), Artesunate Lumefantrine (AL), Dihydroartemisinin-piperaquine (DHP) as first line treatment medications for uncomplicated malaria.


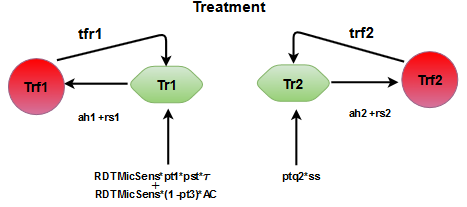


**Fig 1d Model compartments for treatment for uncomplicated and severe malaria cases**

Quinine and other alternatives (intramuscular Artesunate and Artemeter), are administered for severe malaria and cases of treatment failure with first line medicines for uncomplicated malaria.SP on the other hand is given to pregnant women, for prevention of malaria infection, of more than 13 weeks gestation, who attend and are registered at the ANC [8]. Therefore, to account for a potential impact of adherence to drug administration and resistance for all three types of medications, the following transitions have been incorporated into the model:

- proportions of treatment failure (**Trf1**) and non-adherence (**adh1**) to ACT ,Fig 1d
- proportions of treatment failure (**Trf2**) and non-adherence (**adh2**) to Intramuscular quinine ,Artesunate or Artemeter, Fig 1d
- proportions of treatment failure (**Trf3**) and non-adherence (**adh3**) to SP, Fig 1

Depending on adherence to treatment (**ah1** and **ah2**) and the level of drug resistance (**rs1** and **rs2**) Fig 1e below, a proportion of those seeking treatment may fail to clear their infection after 28 days of treatment and be classified as being infected but probably without symptoms whiles those who successfully recovered of all symptoms and parasitaemia return to being susceptible almost immediately into **Snn**.

**Pregnant women receiving care at Antenatal Clinic (ANC)**

The **Snnp** compartment represents the susceptible pregnant women drawn from the **Snn** compartment as described earlier. From **Snnp**, pregnant women are exposed by the same force of infection $\boldsymbol{\lambda}_{\boldsymbol{m}}$ into the latent stage, **L3** from where they make a transition either into **Ic,** if the infection becomes symptomatic, or **Ia** or **Ism,** if the infection is asymptomatic and detectable or undetectable respectively.


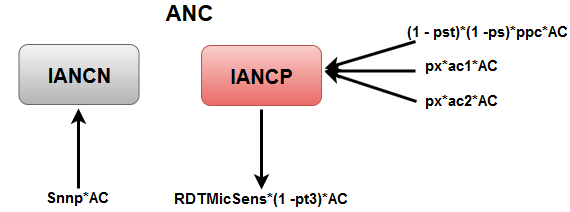


**Fig 1e Model compartments for infectious ANC attendees**

Pregnant women are encouraged to attend Antenatal Clinic (ANC) once monthly after registration from weeks 12-13 gestation [9]. This routine is captured in compartments **IancN** and **IancP** for those attending the ANC without an infection (from **Snnp**) and with an infection (from **Ic**, **Ia** and **Ism**) respectively. Attendees are all screened and tested with an RDT or Microscopy to confirm malaria infection (symptomatic or asymptomatic). From these compartments, a monthly single dose course of Sulfadoxine -pyrimethamine (SP) is administered as a preventative treatment for malaria infection for all pregnant women seen except for those confirmed to be symptomatically infected who are consequently administered an appropriate treatment according to national treatment guidelines for pregnant women [10]. Confirmed clinical cases among pregnant women who get treated are represented by **RDTMicSens*(1 –pt3)*AC** (added to those treated at the Outpatients, as shown in Fig 1d), where **pt3** is the proportion of ANC attendees who receive SP with or without an asymptomatic infection and **AC** is rate of ANC attendance and **RDTMicSens** is the average sensitivity of RDT and Microscopy [11].

Similarly, depending on the level of SP resistance (**rs3**), a proportion of the pregnant women treated in the **Tr3** compartment with SP may fail to prevent an infection and progress into the **Trf3** compartment from where they could subsequently return into **Tr3** compartments for another treatment dose during their next visit if their infection did not develop into clinical malaria.

It is to be noted that, Intermittent Preventive Treatment in pregnancy (IPTp) is primarily to improve birth comes by preventing malaria infections during pregnancy, but since pregnant women presenting at the ANC are screened for clinical malaria infection, among others, and treated appropriately and captured into the ANC register as part of the morbidity cases in the routine data collection on the DHIMS, this process is deemed important and therefore could not to be ignored in the model setup [12].

**Malaria attributable deaths**

Malaria attributable deaths are drawn from the **Isd** compartments only as the deaths due to uncomplicated malaria when treated promptly is insignificant (0.1%). Proportion of under 5 years old and adults were adjusted for and calibrated with estimates from the Global Burden of disease (GBD) [13,14].

**Immunity**

The stable nature of transmission and the variation in seasonality across all three zones requires incorporating superinfection, acquired immunity, treatment failure and seasonality into to the model structure so as to account for the natural history of malaria as much possible that allows for the description of the transmission dynamics of malaria across Ghana.

The models do not incorporate levels of immunity following length of exposure based on age, aspects of the model structure account for this concept even though not fully. Thus the transitions accounting for some level of immunity in the model are:

Children born naïve or young children with little exposure to malaria infection

- - - born into the **Sn** compartment or
    - born with a congenital infection of malaria into the **Ia** compartment
- Adults in the population with several years of exposure
  - - recruited into the **Snn** compartment
- Progressing from **Ism** to **Ic**
- Progressing from **Ic** to **Ia**
- Progressing from **Ia** to **Ism**
- Recovering naturally without treatment from **Ism** to **Snn** [15] ; a state of susceptibility where one is more likely to have an asymptomatic episode of malaria.

**Superinfection**

The models allow for the infected population to get reinfection or superinfection. This is implemented through a superinfection factor that is dependent on the inverse of the sum of the rate of force of infection from mosquito to human ( $\boldsymbol{\lambda}_{\boldsymbol{m}}$ ) and duration of infection ( $\boldsymbol{\gamma}$ ) i.e (1/$\lambda_{m}$+1/$\gamma$)^-1^ _._ This affects the populations in the infected compartments **Ia**, and **Ism**. A proportion of the infected and superinfected therefore make the following transitions:

- progressing from asymptomatic stage **(Ia)** to symptomatic **(Ic)**
- progressing from sub-microscopic **(Ism)** to symptomatic **(Ic)**

**Vector model**

The mosquito population was explicitly modelled relying on vector parameters as shown on Table 2. The vector compartments are the **Lv**, **Sm**, **Em** and **Im** respectively representing the young mosquitoes (larva, pupa), susceptible, exposed and infectious mosquitoes. The susceptible mosquitoes are populated through maturing larva and pupa compartment, **Lv**. The egg deposition rate $\boldsymbol{\emptyset}$and maturing rate ***θ*** are all dependent on the carrying capacity (**Kv**) of the environment to support breeding which in turn depends on water and environmental temperature (Temp) and rainfall (R_f_) [2].


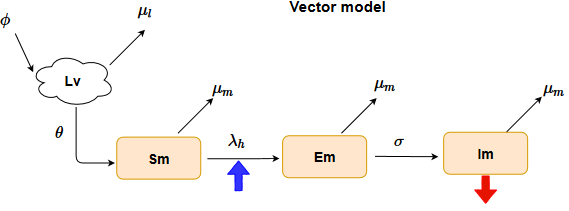


**Fig 1f Vector model compartments**

Therefore the governing functions for the transitions between **Lv**, **Sm**, **Em** and **Im** as captured in Fig 1f and defined on Table 2 below, are temperature dependent as the rate of feeding of matured mosquitoes and of maturing of gametocytes are also temperature dependent [16,17] . Whiles $\boldsymbol{\lambda}_{\boldsymbol{h}}$, is the force of infection (indicated by the blue arrow) of the human population on the susceptible mosquitoes, $\boldsymbol{\mu}_{\boldsymbol{l}}$ and $\boldsymbol{\mu}_{\boldsymbol{m}}$ are the mortality rates for the immature mosquitoes and matured mosquitoes respectively, Fig 1f. The dynamics of the mosquito population for the various zones are shown on Fig 8 in Appendix C.

**Table 1 Definition of state variables**

| **No** | **Variable name** | **Description** |
| --- | --- | --- |
| **1** | **S_hn_** | susceptible naïve |
| **2** | **S_hnn_** | susceptible non-naïve |
| **3** | **S_hnnp_** | susceptible non-naïve pregnant women |
| **4** | **Ib_1h_** | naïve pop: infected/incubation period/potentially infectious/asymptomatic/not detectable by MC |
| **5** | **Ib_2h_** | non-naïve pop: infected/incubation period/ potentially infectious/asymptomatic/not detectable by MC |
| **6** | **Ib_3h_** | non-naïve pregnant women: infected/incubation period/ potentially infectious/asymptomatic/not detectable by MC |
| **7** | **IancN** | pregnant women attending ANC without an infection |
| **8** | **IancP** | pregnant women attending ANC with an infection |
| **9** | **Tr_3h_** | treatment with SP |
| **10** | **Trf_3h_** | treatment failure with SP |
| **11** | **Ic_h_** | infected/ infectious/symptomatic/detectable by MC |
| **12** | **Tr_1h_** | treatment with ACTs |
| **13** | **Trf_1h_** | treatment failure with ACTs |
| **14** | **Ia_h_** | infected/infectious/asymptomatic/detectable by MC |
| **15** | **Ism_h_** | infected/potentially infectious/asymptomatic/sub-microscopic infection/not detectable by MC |
| **16** | **Isd_h_** | infected/ infectious/symptomatic/severe disease/detectable by MC |
| **17** | **Tr_2h_** | treatment with QUININE |
| **18** | **Trf_2h_** | treatment failure with QUININE |
| **19** | **L_v_** | mosquito larva and pupa (immature mosquitoes) population |
| **20** | **S_m_** | mosquitoes susceptible to human infection |
| **21** | **E_m_** | infected/not infectious mosquitoes after a blood meal/biting from an infectious human |
| **22** | **I_m_** | gametocytes develop to become sporozoites thus making mosquitoes infected and infectious |

**Base model equations**

**Human population**

$\frac{\mathrm{dS}_{\mathrm{hn}}}{\mathrm{dt}}=N_{h}{*b}_{h}*p_{n}-{m_{1}*\lambda}_{\mathrm{mh}}*S_{\mathrm{hn}}-\mu_{h}*S_{\mathrm{hn}}$

$\frac{\mathrm{dS}_{\mathrm{hnn}}}{\mathrm{dt}}=N_{h}*b_{h}*\left( 1- p_{n} \right)*p_{m}+{{\left( 1-\left( {ah}_{1}+ {rs}_{1} \right) \right)*rho}_{1}*Tr1}_{h}+\left( 1-\left( {ah}_{2}+ {rs}_{2} \right) \right)*{\mathrm{rho}_{2}*Tr2}_{h}+nr{*I}_{\mathrm{sm}}{+ hlsp*Tr3}_{h}-{m_{1}*\lambda}_{\mathrm{mh}}*S_{\mathrm{hnn}}-px*S_{\mathrm{hnn}}-\mu_{h}{*S}_{\mathrm{hnn}}$

$\frac{\mathrm{dL}_{1h}}{\mathrm{dt}}={m_{1}*\lambda}_{\mathrm{mh}}{*S}_{\mathrm{hn}}-\mathrm{pc}_{1}*\gamma*L_{1h}-\left( 1- \mathrm{pc}_{1} \right)*pa_{1}*\gamma*L_{1h}-\left( 1- \mathrm{pc}_{1} \right)*\left( 1-pa_{1} \right)*\gamma*L_{1h}-\mu_{h}*L_{1h}$

$\frac{\mathrm{dL}_{2h}}{\mathrm{dt}}={m_{2}*\lambda}_{\mathrm{mh}}*S_{\mathrm{hnn}}-\mathrm{pc}_{2}*\gamma*L_{2h}-\left( 1- \mathrm{pc}_{2} \right)*\mathrm{pa}_{2}*\gamma*L_{2h}-\left( 1- \mathrm{pc}_{2} \right)*\left( 1- \mathrm{pa}_{2} \right)*\gamma*L_{2h}-\mu_{h}*L_{2h}$

$\frac{\mathrm{dIc}_{h}}{\mathrm{dt}}=\mathrm{pc}_{1}*\gamma*L_{1h}+\mathrm{pc}_{2}*\gamma*L_{2h}+ppc*\gamma*L_{3h}+q*\mathrm{Ia}_{h}+ superInf*\mathrm{Ia}_{h} + superInf*\mathrm{Ism}_{h}- \mathrm{pt}_{1}*\tau_{1}*pst*{RDTMicSens*Ic}_{h}- \left( 1-pst \right)*ps* ss*\mathrm{Ic}_{h}- \left( 1- pst \right)*\left( 1- ps \right)*\left( 1- ppc \right)*\mathrm{Ic}_{h}- \left( 1- \mathrm{pst}_{1} \right)*\left( 1- ps \right)*ppc*AC*\mathrm{Ic}_{h}-\mu_{h}*\mathrm{Ic}_{h}$

$\frac{{dTr1}_{h}}{\mathrm{dt}}=\mathrm{pt}_{1}*\tau_{1}*pst*RDTMicSens*\mathrm{Ic}_{h}+ tfr1*{Trf1}_{h}+ RDTMicSens*AC*\left( 1 - pt3 \right)*IancP- {{\left( 1- \left( {ah}_{1}+ {rs}_{1} \right) \right)*rho}_{1}*Tr1}_{h}- \left( \mathrm{ah}_{1}+\mathrm{rs}_{1} \right)*{Tr1}_{h}- \mu_{h}*{Tr1}_{h}$

$\frac{{dTrf1}_{h}}{\mathrm{dt}}=\left( \mathrm{ah}_{1}+\mathrm{rs}_{1} \right)*{Tr1}_{h}- {tfr1*Trf1}_{h}-\mu_{h}*{Trf1}_{h}$

$\frac{\mathrm{dIa}_{h}}{\mathrm{dt}}=N_{h}*b_{h}\left( 1- p_{n} \right)*\left( 1- p_{m} \right) + \left( 1- \mathrm{pc}_{1} \right)*\mathrm{pa}_{1}*\gamma*L_{1h}+ \left( 1- \mathrm{pc}_{2} \right)*\mathrm{pa}_{2}*\gamma*L_{2h}+ \left( 1-ppc \right)*ppa*\gamma*L_{3h}+\left( 1- pst \right)* \left( 1-ps \right)*\left( 1- ppc \right)*\mathrm{Ic}_{h}+ x_{1}\mathrm{Ism}_{h}- q*\mathrm{Ia}_{h}-v*\mathrm{Ia}_{h}- AC*px*ac1*Ia - superInf*\mathrm{Ia}_{h} \mathbf{-}\mu_{h}*\mathrm{Ia}_{h}$

$\frac{\mathrm{dIsd}_{h}}{\mathrm{dt}}=\left( 1- pst \right)*ps*ss*\mathrm{Ic}_{h}-\mathrm{ptq}_{2}*ss*\mathrm{Isd}_{h}-\mu_{h}*\mathrm{Isd}_{h}$

$\frac{{dTr2}_{h}}{\mathrm{dt}}=\mathrm{ptq}_{2}*ss*\mathrm{Isd}_{h}+ \left( 1-\left( {ah}_{2}+ {rs}_{2} \right) \right)*{\mathrm{rho}_{2}*Tr2}_{h}+{Trf2}_{h}-{\left( \mathrm{ah}_{2}+\mathrm{rs}_{2} \right)*Tr2}_{h}-\mu_{h}*{Tr2}_{h}$

$\frac{{dTrf2}_{h}}{\mathrm{dt}}={\left( \mathrm{ah}_{2}+\mathrm{rs}_{2} \right)*Tr2}_{h}-{Trf2}_{h}-\mu_{h}*{Trf2}_{h}$

$\frac{\mathrm{dIsm}_{h}}{\mathrm{dt}}=v*\mathrm{Ia}_{h}+\left( 1- \mathrm{pc}_{1} \right)*\left( 1-pa_{1} \right)*\gamma*L_{1h}+\left( 1- \mathrm{pc}_{2} \right)*\left( 1- \mathrm{pa}_{2} \right)*\gamma*L_{2h}+\left( 1- ppc \right)*\left( 1- \mathrm{ppa} \right)*\gamma*L_{3h} - x_{1}\mathrm{Ism}_{h}- nr*\mathrm{Ism}_{h}- AC*px*ac2*Ism- superInf*\mathrm{Ism}_{h}-\mu_{h}*\mathrm{Ism}_{h}$

$\frac{dS_{\mathrm{hnnp}}}{\mathrm{dt}}=S_{\mathrm{hnn}}*p_{x}- m_{1}*\gamma{*S}_{\mathrm{hnnp}}-AC*S_{\mathrm{hnnp}}- \mu_{h}*S_{\mathrm{hnnp}}$

$\frac{\mathrm{dL}_{3h}}{\mathrm{dt}}={m_{1}*\lambda}_{\mathrm{mh}}*S_{\mathrm{hnnp}}-ppc*\gamma*L_{3h}-\left( 1- ppc \right)*ppa*\gamma*L_{3h}-\left( 1- ppc \right)*\left( 1-ppa \right)*\gamma*L_{3h}- \mu_{h}*L_{3h}$

$\frac{\mathrm{dIancN}_{h}}{\mathrm{dt}}=A{C*S}_{\mathrm{hnnp}}-AC*\mathrm{pt}_{3}*\mathrm{IancN}_{h}-\mu_{h}*\mathrm{IancN}_{h}$

$\frac{\mathrm{dIancP}_{h}}{\mathrm{dt}}=\left( 1- pst \right)*\left( 1- ps \right)*ppc*AC*\mathrm{Ic}_{h}+ AC*px*ac1*Ia + AC*px*ac2*Ism - AC*pt3*IancP - RDTMicSens*AC* (1 - pt3)*IancP-\mu_{h}*\mathrm{IancP}_{h}$

$\frac{{dTr3}_{h}}{\mathrm{dt}}=AC*\mathrm{pt}_{3}*\mathrm{IancN}_{h}+AC*\mathrm{pt}_{3}*\mathrm{IancP}_{h}+AC*{Trf3}_{h}-{\left( \mathrm{ah}_{3}+\mathrm{rs}_{3} \right)*Tr3}_{h}-hlsp*{Tr3}_{h}- \mu_{h}*{Tr3}_{h}$

$\frac{{dTrf3}_{h}}{\mathrm{dt}}={\left( \mathrm{ah}_{3}+\mathrm{rs}_{3} \right)*Tr3}_{h}- AC*{Trf3}_{h}-\mu_{h}*{Trf3}_{h}$

**Mosquito population**

$\frac{\mathrm{dL}_{v}}{\mathrm{dt}}=\emptyset_{v}*\left( 1- \frac{L_{v}}{Kv*\left( Rf+1 \right)} \right)*\left( S_{m}+ E_{m}+ I_{m} \right)-\theta_{v}*L_{v}-\mu_{l}*L_{v}$

$\frac{\mathrm{dS}_{m}}{\mathrm{dt}}=\theta_{v}*L_{v}-\lambda_{\mathrm{hm}}*S_{m}-\mu_{m}*S_{m}$

$\frac{\mathrm{dE}_{m}}{\mathrm{dt}}=\lambda_{\mathrm{hm}}S_{m}- \sigma_{v}{*E}_{m}- \mu_{m}{*E}_{m}$

$\frac{\mathrm{dI}_{m}}{\mathrm{dt}}=\sigma_{v}{*E}_{m}- \mu_{m}{*I}_{m}$

**Table 2: Table of Parameters and derived equations used in the vector model**

| Parameter | Equation | Definition | Units | Source |
| --- | --- | --- | --- | --- |
| $\emptyset_{v}$,EFD | $-0.153* {Temp}^{2}+8.61*Temp - 97.7$ | Temperature dependent egg deposition rate, Number of eggs laid per female mosquito | days^-1^ | [16] |
| $\mathrm{PEA}$ | $-0.00924* \hat{Temp}^{2}+ 0.453*\hat{Temp}- 4.77$ | Probability an egg survives to adult mosquito | - | [16] |
| $\tau_{\mathrm{EA}}$ | $\frac{1}{(-0.00094*\hat{Temp}^{2} + 0.049*\hat{Temp}-0.552)}$ | Development time from egg to adult mosquito | days^-1^ | [16] |
| $\mu_{l}$ | $\frac{1}{(8.560 + 20.654*\left[ {1 + \left( \frac{\hat{Temp}}{19.759} \right)}^{6.827} \right]^{(-1)}}$ | Temperature dependent natural mortality rate of immature vectors | days^-1^ | [16] |
| $\mu_{m}$ | $\left( -\log\left( -0.000828*{Temp}^{2} + 0.0367*Temp + 0.522 \right) \right)$ | Temperature dependent natural mortality rate of adult vectors | days^-1^ | [16] |
| $\sigma_{v}$ | $-0.00083*{Temp}^{2} + 0.044*Temp - 0.487$ | Temperature dependent progression rate of exposed vectors | days^-1^ | [16] |
| $\mathrm{BT}$ | $\frac{\mathrm{EFD}}{\mu_{m}}$ | Lifetime number of eggs laid | - | [16] |
| $\theta_{v}$ | $\frac{BT*PEA}{\tau_{\mathrm{EA}}}$ | Temperature dependent maturation rate from eggs to adult mosquito | days^-1^ | [16] |
| $\mathrm{Kv}$ | Environmental carrying capacity of immature mosquitoes (larva + pupa) | | ha^-1^ | [17] |

*^1^Temp : monthly average temperature at the zonal level*

*^2^* $\hat{Temp}$*: monthly average water temperature. Here it is approximated to Temp*

*^3^Rf : monthly mean rainfall at the zonal level*

**Table 3 Parameter values**

| **Parameter** | **Parameter value by Zone** | | | **Parameter definition** | **Source** |
| --- | --- | --- | --- | --- | --- |
| **Name** | **Guinea Savannah** | **Transitional Forest** | **Coastal Savannah** |  |  |
| pc1 | 0.9 | 0.9 | 0.8 | probability of naive progressing into **Ic** | estimated |
| pa1 | 0.35 | 0.07 | 0.58 | probability of naive progressing into **Ia** | estimated |
| pc2 | 0.14 | 0.19 | 0.14 | probability of non-naive progressing into **Ic** | estimated |
| pa2 | 0.61 | 0.39 | 0.49 | probability of non-naive progressing into **Ia** | estimated |
| ps | 0.13 | 0.065 | 0.062 | probability of progressing into severe disease | [18] |
| pt1 | 0.87 | 0.88 | 0.88 | probability of being tested/diagnosed for uncomplicated malaria | [19] |
| ppc | 0.81 | 0.7 | 0.8 | proportion of pregnant women from **L3** progressing to **Ic** | estimated |
| ppa | 0.25 | 0.075 | 0.54 | proportion of pregnant women from **L3** progressing to **Ia** | estimated |
| x | 0.01 | 0.01 | 0.01 | probability of progressing from **Ia** to **Ic** | estimated |
| m1 | 0.57 | 0.1 | 0.1 | probability of infection among children under 6 years and pregnant women | estimated |
| m2 | 0.77 | 0.22 | 0.2 | probability of infection among non-naive population 6 years and above | estimated |
| pst | 0.8 | 0.71 | 0.73 | probability of seeking treatment at the health facility | [19] |
| prob | 0.5 | | | probability of a bite resulting into a mosquito being infected or a human being infected following a bite from an mosquito | [20] |
| pn | 0.125 | 0.125 | 0.125 | population of children under 6 years (number) | ][21] |
| pm | 0.874375 | 0.87425 | 0.874415 | population 6 years and above (number) | [15] |
| pt2 | 0.99 | 0.99 | 0.99 | probability of being treated with **QUININE** | [22] |
| ah1 | 0.385 | 0.385 | 0.385 | proportion non-adherent to **ACT** treatment | [23] |
| ah2 | 0.092 | 0.082 | 0.082 | proportion non-adherent to **QUININE** treatment | [22,24] |
| px | 0.025 | | | proportion of pregnant women in the population | [21] |
| rs1 | 0.04 | 0.04 | 0.04 | resistance against **ACT** (Day 28 PCR-corrected failure rate (0.8%- 4.0%) for ASAQ and AL **(**proportion**)** | [25] |
| rs2 | 0.01 | 0.01 | 0.01 | resistance against **QUININE,** intramusclar **ARTEMETHER** (Day 28 parasitaemia failure rate) **(**proportion**)** | [26] |
| rs3 | 0.0962 | 0.0962 | 0.0962 | resistance against **SP** (Day 28 PCR-corrected failure rate (0.0962) for SP **(**proportion**)** | [27] |
| ac1 | 0.134 | 0.126 | 0.112 | probability of asymptomatic malaria among pregnant women at **ANC** | [28–30] |
| ac2 | 0.097 | 0.097 | 0.097 | probability of sub-microscopic infection among pregnant women at **ANC** | [21] |
| pt3 | 0.367 | | | proportion of pregnant women taking up at least 3 dose | [6] |
| ah3 | 0.633 | | | proportion of pregnant women not taking up at least 3 doses | [6] |
| Nn | 5.1 x 10^6^ | 17.1 x 10^6^ | 8.1 x 10^6^ | human population size (2018 mid-year estimated) (number) | DHIMS2 |
| Ln | 25/1000 | 30.6/1000 | 23.5/1000 | birth/death rate per 1000 population (year ^-1^) | [31–33] |
| Kv | 7.8 x10^5^ | 4.2 x 10^7^ | 2.5 x 10^7^ | carrying capacity of the environment for larva and pupae stages of mosquitoes (ha^-1^) | estimated |
| LLIN | 0.398 | | | protective efficacy of **LLINs** against malaria (based on the IRR^#^ or OR^##^) | [34] |
| IRS | 0.285 | | | protective efficacy of **IRS** against malaria (based on the IRR^#^ or OR^##^) | [34] |
| ss | 365.25/5 | | | rate of progressing into severe disease(days^-1^) | [35] |
| q | 365.25/194 | | | duration of progressing from **Ia** into **Ic** (days^-1^) | [23,36,37] |
| gamma | 365.25/21 | | | duration of latent period in human population(days^-1^) | [38] |
| t1 | 365.25/3 | | | duration after onset of illness **ACT** treatment was sought (days^-1^) | [38] |
| rho1 | 365.25/3 | | | recovery rate after ACT treatment(days^-1^) | [38,39] |
| rho2 | 365.25/6 | | | recovery rate after **QUININE** treatment(days^-1^) | [40] |
| v | 52/5.5 | | | rate of recovery from **Ia** to **Ism** without treatment(weeks^-1^) | [41] |
| nr | 365.25/130 | | | rate of natural recovery from infection(days^-1^) | [42] |
| AC | 365.25/30 | | | rate of antenatal attendance(days^-1^) | [9] |
| hlsp | 365.25/8 | | | rate of recovering after **SP** treatment at **ANC**(days^-1^) | [43] |
| RDTMicSens | 0.49 | | | average sensitivity of **RDTs** and Microscopy in health facilities (proportion) | [11] |

*^#^ IRR –Incidence rate ratio*

*^##^ OR-Odds ratio*

**Force of infection**

Transmission of malaria parasites between humans and mosquitoes is through the draw of a blood meal from humans by infectious mosquitoes. The likelihood of humans being infected upon a successful bite of a mosquito will to some extent depend on the level of susceptibility of getting infected. On the other hand a non-infected mosquito drawing a blood meal from an infected human also has a probability of ingesting gametocytes which later develop into sporozoites.

In this model, a 50% chance of transmitting the malaria parasite between humans and mosquitoes following a successful bite of an infected mosquito on humans or an uninfected mosquito on humans in any of the infected stages was considered [20].

The forces of infections are defined as:

| $\boldsymbol{\lambda}_{\mathbf{hm}}= prob\_inf*(1- ITNcov*ITNeff*ITNusage)*(1- IRScov*IRSeff)*BR*\frac{\left( \mathrm{Ic}_{h}+\mathrm{Ia}_{h}+\mathrm{Isd}_{h}+\mathrm{Ism}_{h}+\mathrm{IancP}_{h}+{Tr1}_{h}+{Tr2}_{h}+{Tr3}_{h}+{Trf1}_{h}+{Trf2}_{h}+{Trf3}_{h} \right)}{N_{h}}$ | | (1) | |
| --- | --- | --- | --- |
| $\boldsymbol{\lambda}_{\mathbf{mh}}\mathbf{=}prob\_inf*(1- ITNcov*ITNeff*ITNusage)*(1- IRScov*IRSeff)*BR*\frac{I_{m}}{N_{h}}$ | (2) | |  |

where Equation (1) represents the force of infection from humans to mosquitoes likewise with Equation (2) for the force of infection from mosquitoes to humans. The contact rate is represented by the Biting Rate (BR). The BR data were obtained from field studies from each of the zones through human landing catches (HLC). They are defined as the average number of bites received by a human in the population per month (b/p/m) as shown on Fig 2a, 2b, 2c respectively [44,45].

The probability that, following a bite, sporozoites or gametocytes from infected humans may be transmitted or transferred to mosquitoes of from infected mosquitoes to humans respectively is represented by **prob_inf**.

The fraction of the population of infected humans carrying sporozoites, with a 50% probability of infecting a mosquito upon a draw of a blood meal, we considered to be contained in the compartments $\frac{\left( \mathbf{Ic}_{\mathbf{h}}\mathbf{+}\mathbf{Ia}_{\mathbf{h}}\mathbf{+}\mathbf{Isd}_{\mathbf{h}}\mathbf{+}\mathbf{Ism}_{\mathbf{h}}\mathbf{+}\mathbf{IancP}_{\mathbf{h}}\mathbf{+}\mathbf{Tr1}_{\mathbf{h}}\mathbf{+}\mathbf{Tr2}_{\mathbf{h}}\mathbf{+}\mathbf{Tr3}_{\mathbf{h}}\mathbf{+}\mathbf{Trf1}_{\mathbf{h}}\mathbf{+}\mathbf{Trf2}_{\mathbf{h}}\mathbf{+}\mathbf{Trf3}_{\mathbf{h}} \right)}{\mathbf{N}_{\mathbf{h}}}$ as in Equation (1) and similarly the fraction of the mosquito population considered to be carrying gametocytes that could potentially be passed on to the blood stream of a human victim is also represented by $\frac{\mathbf{I}_{\mathbf{m}}}{\mathbf{N}_{\mathbf{h}}}$. [20].

Coverage levels and effectiveness of ITNs and IRS are denoted by *itnc(t)* = (1 - *itncov*itneff*itnusage*) and *irsc(t)* = (1 - *irscov***irseff*) respectively, where *itncov* and *irscov* and *itneff* and *irseff* represent coverage levels and effectiveness for both ITNs and IRS respectively with time and *itnusage* is level of ITN/LLIN usage.

**Data fitting and Model calibration**

The model was fitted with zone specific monthly confirmed uncomplicated malaria cases, severe malaria cases and malaria among pregnant women. Data captured from 2008 to 2011 (DHIMS1) and 2012 to 2017 (DHIMS2) were used. The pattern of the data from 2008 to 2017 seem to suggest an increasing trend in the incidence of malaria in Ghana. However as pointed out elsewhere, these seeming increasing trend is largely due to reporting, increasing diagnostic testing and perhaps improvement in the health seeking [2]. The data fitting process was therefore also adjusted for using reporting probabilities of the health facilities capturing all confirmed cases of malaria onto the DHIMS2 platform.

Universal household coverage levels of ITNs/LLINs and IRS were incorporated in the model build up and a reporting probability of patients to the health facility as well as the gradual capacity improvement of the DHIMS to capture more data were also included.

As shown on Fig 3 in Appendix A, the models were evaluated for ten years from 1988 to 1998 so as to allow it to reach equilibrium state. Estimated prevailing ITN and IRS coverage levels (Table 4) were then adjusted for from 2008 to 2017.

**Table 4 LLIN and IRS coverage levels by zone**

| **years** | **LLIN coverage (%)** | | | **IRS coverage (%)** | | | **Source** |
| --- | --- | --- | --- | --- | --- | --- | --- |
|  | **Guinea**  **savannah** | **Transitional forest** | **Coastal**  **savannah** | **Guinea**  **savannah** | **Transitional**  **forest** | **Coastal savannah** |  |
|  |  |  |  |  |  |  |  |
| 2008 | 0.29 | 0.22 | 0.12 | 0 | 0 | 0 | [46] |
| 2009 | 0.6 | 0.43 | 0.38 | 0.18 | 0 | 0 |  |
| 2010 | 0.6 | 0.43 | 0.38 | 0.18 | 0 | 0 |  |
| 2011 | 0.6 | 0.43 | 0.38 | 0.18 | 0 | 0 | [47] |
| 2012 | 0.6 | 0.64 | 34 | 0.18 | 0.04 | 0 |  |
| 2013 | 0.6 | 0.64 | 0.34 | 0.18 | 0.04 | 0 |  |
| 2014 | 0.39 | 0.53 | 0.41 | 0.13 | 0 | 0 | [48] |
| 2015 | 0.39 | 0.53 | 0.41 | 0.13 | 0 | 0 |  |
| 2016 | 0.66 | 0.51 | 0.5 | 0.17 | 0 | 0 | [19] |
| 2017 | 0.66 | 0.51 | 0.5 | 0.17 | 0 | 0 |  |

Data fitting therefore started from 2008 to 2017 and predictions made within the period 2018 to 2030. Various intervention scenarios were tested within the prediction period from 2018 to 2030 to ascertain their effectiveness in averting potential malaria cases across the different zones.

Given the high dimensionality of the monthly aggregated counts of confirmed multiple categories of malaria cases from each ecological zone, direct parameter estimation through the computation of the likelihood was intractable or laborious if possible. For this reason, an Approximate Bayesian Computation (ABC) approach was adopted for model calibration.

Based on the Bayesian paradigm, estimates of the posterior distribution are generated through a stochastic sampling of the prior parameter distribution. This process affords the avoidance of calculating the likelihood function but enables estimation of model parameters for all three zones [49,50].

In the Bayesian statistics framework, consider that the data (***D***), given a set of parameters (θo) , generated by the model (***M***) is denoted by

$p\left( \theta_{o}|\mathbf{D} \right)= \frac{p\left( {\mathbf{D}|\theta}_{o} \right)*p\left( \theta_{\pi} \right)}{p\left( \mathbf{D} \right)}$ (3)

Where

$p\left( \mathbf{D} \right)=\int p\left( {\mathbf{D}|\theta}_{o} \right)*p\left( \theta_{\pi} \right)d\theta_{o}$ (4)

and $\theta_{\pi}$ are priors or initial knowledge of the parameters of the model. Since Equation (4) is a normalising constant and $p\left( \theta_{o}|\boldsymbol{D} \right)$ is the posterior density and given that $p\left( {\boldsymbol{D}|\theta}_{o} \right)$ is the likelihood, then the posterior density is proportional to the likelihood through

$p\left( \theta_{o}|\mathbf{D} \right) \propto p\left( {\mathbf{D}|\theta}_{o} \right)*p\left( \theta_{\pi} \right)$ (5)

Equation (5) is evaluated stochastically following the steps below once summary statistic(s) is chosen for the observed data:

1. Evaluate statistics for observed data *D_obs_* to get observed summary statistic, *S_obs_*
2. Sample θ from the distribution of the priors$p\left( \theta_{\pi} \right)$.
3. Simulate *D_sim_* from the model ***M*** with sampled parameters θ.
4. Apply the chosen statistic on the simulated data *D_sim_* to obtain *S_sim_*
5. Calculate a distance metric ***d*** (*S_sim_* , *S_obs_*) between *S_sim_* and *S_obs_*.
6. Accept θ if ***d*** <= ***ε*** and return to *1.* [49,51,52]

**The distance metric**, ***d*** (*S_sim_*, *S_obs_*)

The distance metric ***d,*** for each iteration of the models were calculated based on **Equation (6)**, adopted from [53]. The whole vector of the observed data for each of the five datasets ,namely *uncomplicated malaria* ,*severe malaria*, *malaria in pregnancy*, *malaria attributable deaths for children under five and adults* were used in place of summary statistics as outlined in (1) above. Similarly each vector of simulation output was used in place of a simulation summary statistic.

As shown in **Equation (6)**, the distance ***d*** between each simulation output ***i,*** $S_{sim,i}$, for dataset ***j*** and $S_{obs,j}$, the observed data vector for dataset ***j*** is calculated and standardised by ${sd(S}_{sim,j})$, the standard deviation of simulations arising from dataset ***j***, where ***i***=1,2…….120 months and ***j***=1,2,…5, datasets. Standardising with the standard deviation was carried out because of the varied scales of measurement of the five datasets that needed to be fitted simultaneously.

$d\left( S_{sim,ij}-S_{obs} \right)=\sqrt{\sum_{j} \left( \frac{S_{sim,ij}-S_{obs,j}}{sd({S_{sim,}}_{j})} \right)^{2}}$ (6)

At least fifteen thousand (15,000) simulations were performed on the University of Cape Town (UCT) High Performance Computing Cluster (HPC) ([http://hpc.uct.ac.za](http://hpc.uct.ac.za)) for each of the models (Guinea savannah, Transitional forest and Coastal savannah).Based on the ordered calculated distances for all five datasets of each model, a tolerance level of ***ε*** which returned at least a sample of a 100 simulated parameter values, which simultaneously minimised the distances between the observed datasets and the simulated datasets for each model, were retained.

**Validation of model parameters**

The accuracy of parameters were estimated through a validation process carried out using the R package **cv4abc** using the sample parameters that were retained with respect to the distance criteria of **Equation (6).** The package implements a leave-one-out cross validation algorithm and calculates the prediction error for each parameter and their sensitivity or robustness to various tolerance levels [54].

The plots shown in Appendix D present the medians of the posterior distribution of each parameter for each validation sample based on varied tolerance levels depending on the model using the rejection method. The diagonal line in each plot indicates the identity of both the estimated and the true parameter values. Clustering of the median estimates and true parameter values of the cross-validation samples for two tolerance levels around the identity line suggests low predictive errors as well as a high level of precision to varying tolerance levels of the parameter. As shown, each of the three models estimated most of the parameters fairly accurately with low predictive errors and high precision [Appendix D].

**Sensitivity analyses**

Results shown on table’s 5, 6 and 7, in Appendix E were obtained from a multivariate regression based sensitivity analyses of model parameters for each zone. Included in the tables are an ordered set of the standardised coefficients of the parameters in a multivariate regression of the samples obtained in the ABC. The most sensitive parameters vary for each model. In the Guinea savannah model, the most sensitive parameters are ***hlsp, ptq2, prob_hm, pt3*** *and* ***ah1*** in that order whereas for the Transitional forest and Coastal savannah models ***hlsp, h, v, prob_hm, rho1*** and ***hlsp, h, v, prob_hm, pc2*** are most sensitive in that order respectively.

**Interventions**

Interventions were tested from 2018 to 2030.This was to allow for the investigation of the impact of various interventions singly or in combination on the incidence of malaria by various set targets within this time frame.

The various scenarios for LLINs and IRS were investigated, for each intervention and combination, in each zone:

1. Rolling out Only LLIN to achieve a universal coverage within three years at
   1. 70%,80% and 90% and at protective efficacy of 40% at a varied scaled-up usage levels of 60%,80% and 90% from reported levels of usage [55]
2. Implementing only IRS for a period of five years to achieve coverages of
   1. 80% and 90% with a varied level of protective efficacy of 30%,60% and 80%
3. Combined implementation of LLINs and IRS for three years and five years respectively to attain
   1. 80% LLINs and 80% IRS at baseline LLIN usage and protective efficacies of 40% and 30% respectively
   2. 80% LLINs and 80% IRS at baseline LLIN usage and protective efficacies of 60% and 30% respectively
   3. 80% LLINs and 90% IRS at baseline LLIN usage and protective efficacies of 40% and 30% respectively
   4. 90% LLINs and 80% IRS at baseline LLIN usage and protective efficacies of 40% and 30% respectively
   5. 90% LLINs and 90% IRS at baseline LLIN usage and protective efficacies of 40% and 30% respectively
4. SMC among children less than 6 years in the savannah zone was investigated using a modified base model as shown in Fig 2. The children are drawn from all the compartments except those in the treatment and treatment failure compartments, **Tr1**, **Trf1** and **Tr2**, **Trf2** for uncomplicated malaria and severe malaria respectively. The number of number children recruited from the naïve susceptible (**Sn**) children under 6 years of age, non-naïve susceptible (**Snn**), symptomatic (**Ic**), asymptomatic (**Ia**), severe malaria (**Isd**) and sub-microscopic (**Ism**) compartments transit through the **SMC** compartment as shown. The following scenarios were applied:
   1. Treatment with SP +AQ for four rounds (four months) through the rainy season (July to Oct) for children between 3 - 59 months of age.
   2. Sample population of 50% vs 90% in each round


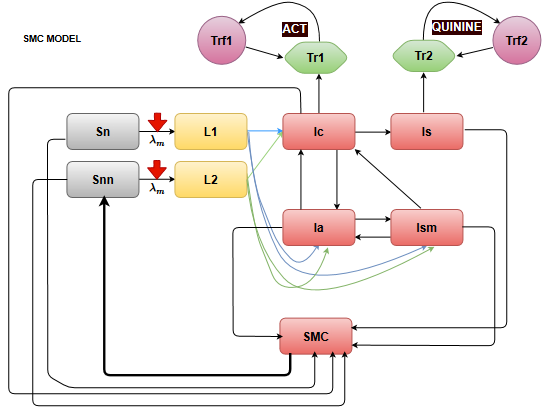


**Fig 2 Sub model for investigating Seasonal Malaria Chemotherapy (SMC) in the Guinea savannah zone.**

It is assumed here that, treated children remain protected within the period of treatment which last for a month and assumed to be protected for four months for the four months of treatment in a year.

1. The impact of MSAT under various scenarios were also investigated in the forest and coastal zones as depicted by Fig 3, which is built into the base model in Fig 1. With respect to MSAT, only sampled members of the population who tested positive for malaria infection with an RDT were considered for treatment. The following scenarios were applied:
   1. Treatment with ACTs four times a year. Two in May and June in the major raining season and two in Sept and Oct in minor raining season
   2. Sample population of 20% vs 50% in each round


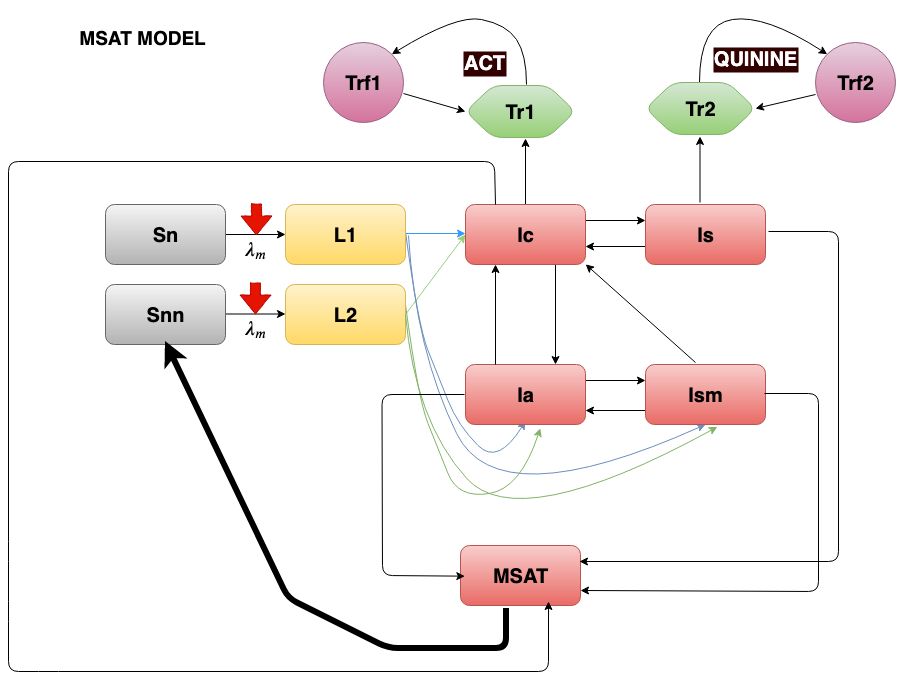


**Fig 3 Sub model for investigating Mass Screen and Treat (MSAT) in the Transitional forest and Coastal savannah zones.**

**Appendix A:** Graphs for the dynamics of human population model with fitted parameter values

**Guinea savannah**

**Fig 4a: Model fit to data for reported uncomplicated case for the Guinea savannah zone.**

**Fig 4b: Model fit to data for reported severe cases for the Guinea savannah zone.**

**Fig 4c: Model fit to data for reported malaria in pregnancy for the Guinea savannah zone.**

**Transitional forest**

**Fig 5a: Model fit to data for reported uncomplicated case for the Transitional forest zone.**

**Fig 5b: Model fit to data for reported severe cases for the Transitional forest zone.**

**Fig 5c: Model fit to data for reported malaria in pregnancy for the Transitional forest zone.**

**Coastal savannah**

**Fig 6a: Model fit to data for reported uncomplicated case for the Coastal savannah zone**.

**Fig 6b: Model fit to data for reported severe cases for the Coastal savannah zone**.

**Fig 6c: Model fit to data for reported malaria in pregnancy for the Coastal savannah zone**.

**Appendix B: Biting Rates (BR) (b/p/m) and rainfall (mm) by zone**

**
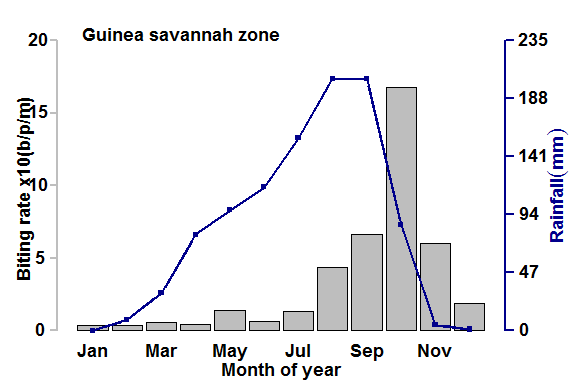
**


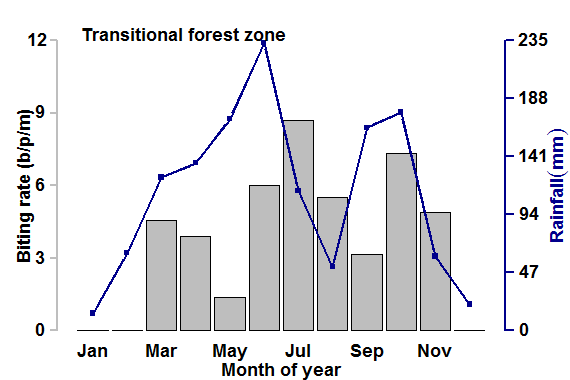


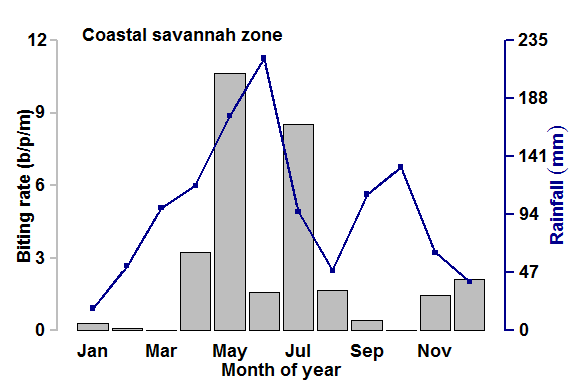


**Fig 7: Monthly biting rates (b/p/m) [Grey Bars] and rainfall (mm) [Blue Lines] in the Guinea savannah, Transitional forest and Coastal savannah.**

**Appendix C:** Dynamics of immature mosquito population by zone

**Guinea savannah**


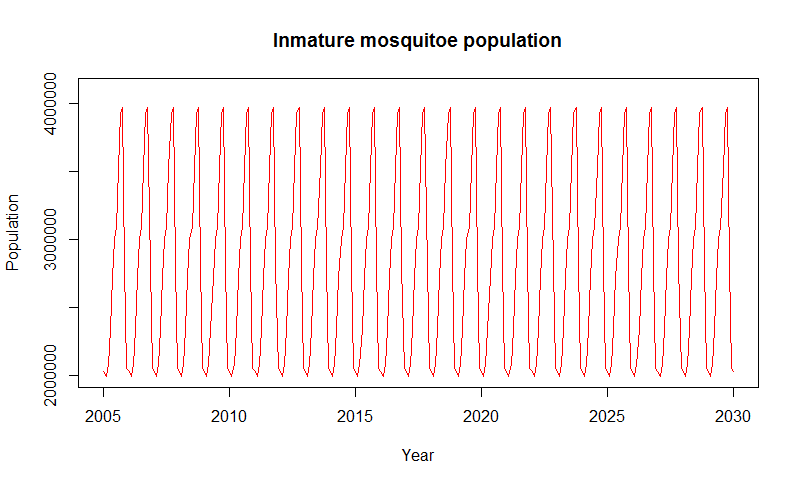


**Fig 8a: Immature mosquito (Larva and pupa) population dynamics for Guinea savannah zone.**

**Transitional forest**  **
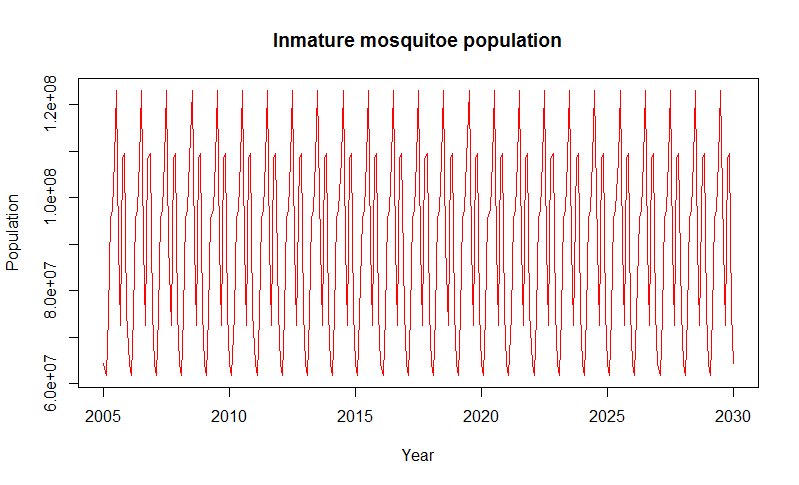
**

**Fig 8b: Immature mosquito (Larva and pupa) population dynamics for Transitional forest zone.**

**Coastal savannah**


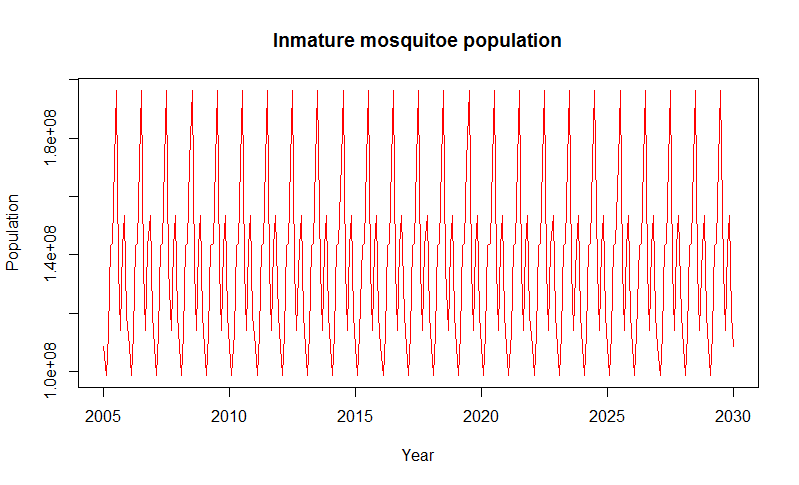


**Fig 8c: Immature mosquito (Larva and pupa) population dynamics for Coastal savannah zone.**

**Appendix D:**

**Guinea savannah**

 **Fig 9a: Graphs for prediction errors [with tolerances of 0.01 (red points) and 0.02 (blue points)] for model parameters estimated by the Approximate Bayesian Computation (ABC) for Guinea savannah zone.**

**Transitional forest**

** Fig 9b: Graphs for prediction errors errors [with tolerances of 0.01 (red points) and 0.02 (blue points)]for model parameters estimated by the Approximate Bayesian Computation (ABC) for Transitional zone.**

**Coastal savannah**

**Fig 9c: Graphs for prediction errors errors [with tolerances of 0.01 (red points) and 0.02 (blue points)] for model parameters estimated by the Approximate Bayesian Computation (ABC) for Coastal savannah zone.**

**Appendix E:**

**Table 5: Sensitivity analysis for parameters of the Guinea savannah model**

| **Guinea savannah** | | | | | |
| --- | --- | --- | --- | --- | --- |
| **Multiple regression estimation** | | |  | **Standardised and ordered coefficients** | |
| **Parameter** | **Estimate** | **P-value** |  | Parameter | coefficient |
| (Intercept) | 12740000.00 | 0.7598 |  | **hlsp** | **9.41** |
| pc1 | 3934000.00 | 0.0292 |  | **ptq2** | **1.14** |
| pa1 | 323600.00 | 0.394 |  | **pro_hm** | **0.62** |
| pc2 | 84090000.00 | < 2e-16 |  | **pt3** | **0.60** |
| pa2 | 2244000.00 | 5.84E-07 |  | **ah1** | **0.56** |
| ppc | 6772000.00 | 0.2667 |  | pc2 | 0.34 |
| ppa | 167000.00 | 0.8369 |  | m2 | 0.21 |
| x | -92100000.00 | 0.6131 |  | pst | 0.14 |
| Kv | -70.81 | 0.1706 |  | q | 0.14 |
| m1 | 4064000.00 | < 2e-16 |  | ac1 | 0.14 |
| m2 | 15870000.00 | < 2e-16 |  | tfr1 | 0.11 |
| ps | 1615000.00 | 8.06E-06 |  | ss | 0.09 |
| pst | 2656000.00 | 0.4535 |  | m1 | 0.07 |
| pn | 68070000.00 | < 2e-16 |  | ps | 0.05 |
| pm | 757800.00 | 0.0356 |  | pa2 | 0.04 |
| ss | -2417000.00 | 2.55E-11 |  | pc1 | 0.02 |
| h | -4446000.00 | < 2e-16 |  | nr | 0.01 |
| q | -282800.00 | 0.4356 |  | Kv | 0.01 |
| gamma | 6910000.00 | 0.0582 |  | ppc | 0.01 |
| t1 | 2081000.00 | 0.7746 |  | rho1 | 0.01 |
| rho1 | -15730.00 | 0.2515 |  | pa1 | 0.01 |
| ptq2 | -13860.00 | 0.2439 |  | h | 0.01 |
| rho2 | 1880000.00 | 0.3774 |  | gamma | 0.00 |
| v | 119200.00 | 0.337 |  | x | 0.00 |
| nr | -6056.00 | 0.1925 |  | ppa | 0.00 |
| ah1 | -8282.00 | 0.0649 |  | px | 0.00 |
| ah2 | -349000.00 | 0.3406 |  | ac2 | 0.00 |
| px | 22240.00 | 0.2623 |  | pro_mh | 0.00 |
| pt3 | 3500.00 | 0.8331 |  | pn | 0.00 |
| ah3 | -1374000.00 | 0.1533 |  | AC | 0.00 |
| hlsp | -6524.00 | 0.7065 |  | pm | 0.00 |
| pro_hm | 22580000.00 | < 2e-16 |  | ah2 | 0.00 |
| pro_mh | 31040000.00 | < 2e-16 |  | rho2 | 0.00 |
| AC | -52910.00 | 0.8394 |  | v | 0.00 |
| ac1 | 26760.00 | 0.9412 |  | ah3 | 0.00 |
| ac2 | 6876000.00 | < 2e-16 |  | t1 | 0.00 |
| tfr1 | 121500.00 | 0.6443 |  |  |  |

**Table 6: Sensitivity analysis for parameters of the transitional forest model**

| **Transitional forest** | | | | | |
| --- | --- | --- | --- | --- | --- |
| **Multiple regression estimation** | | |  | **Standardised and ordered coefficients** | |
| Parameter | Estimate | P-value |  | Parameter | coefficient |
| (Intercept) | 3149000000.00 | 0.78 |  | **hlsp** | **10.41** |
| pc1 | 3223000.00 | 0.73 |  | **h** | **7.49** |
| pa1 | 50440000.00 | 0.17 |  | **v** | **3.36** |
| pc2 | 314600000.00 | < 2e-16 |  | **pro_hm** | **0.58** |
| pa2 | 20240000.00 | 0.03 |  | **rho1** | **0.37** |
| ppc | -11000000.00 | 0.55 |  | pc2 | 0.33 |
| ppa | 37490000.00 | 0.31 |  | m2 | 0.10 |
| x | 18790000000.00 | 0.04 |  | ac1 | 0.09 |
| Kv | -73.97 | 0.78 |  | pm | 0.07 |
| m1 | 112700000.00 | 0.00 |  | m1 | 0.05 |
| m2 | 247600000.00 | < 2e-16 |  | ah1 | 0.04 |
| ps | -66120000.00 | 0.00 |  | tfr1 | 0.02 |
| pst | -31200000.00 | < 2e-16 |  | pa2 | 0.02 |
| pn | 136100.00 | 0.94 |  | x | 0.02 |
| pm | -3759000.00 | 0.04 |  | pn | 0.02 |
| ss | -45790.00 | 0.51 |  | ps | 0.01 |
| h | 10550.00 | 0.94 |  | pa1 | 0.01 |
| q | -135800000.00 | 0.01 |  | ppa | 0.01 |
| gamma | -513200.00 | 0.42 |  | rho2 | 0.01 |
| t1 | -49060.00 | 0.04 |  | t1 | 0.01 |
| rho1 | 18970.00 | 0.41 |  | AC | 0.01 |
| ptq2 | -1107000.00 | 0.55 |  | ppc | 0.00 |
| rho2 | 15350.00 | 0.88 |  | pro_mh | 0.00 |
| v | -90640.00 | 0.30 |  | ah2 | 0.00 |
| nr | -38130000.00 | 0.12 |  | pc1 | 0.00 |
| ah1 | 7913000.00 | 0.00 |  | nr | 0.00 |
| ah2 | -9475000.00 | 0.80 |  | Kv | 0.00 |
| px | -14610000.00 | 0.70 |  | ss | 0.00 |
| pt3 | -3624000.00 | 0.05 |  | pt3 | 0.00 |
| ah3 | 227900.00 | 0.90 |  | px | 0.00 |
| hlsp | 11480.00 | 0.89 |  | gamma | 0.00 |
| pro_hm | 117100000.00 | < 2e-16 |  | pst | 0.00 |
| pro_mh | 139600000.00 | < 2e-16 |  | ac2 | 0.00 |
| AC | 796500.00 | 0.56 |  | q | 0.00 |
| ac1 | -1063000.00 | 0.56 |  | ptq2 | 0.00 |
| ac2 | 22370000.00 | 0.23 |  | ah3 | 0.00 |
| tfr1 | -1309000.00 | 0.34 |  |  |  |

**Table 7: Sensitivity analysis for parameters of the Coastal savannah model**

| **Coastal savannah** | | | | | |
| --- | --- | --- | --- | --- | --- |
| **Multiple regression estimation** | | |  | **Standardised and ordered coefficients** | |
| Parameter | Estimate | P-value |  | parameter | coefficient |
| Intercept | 292000000.00 | 0.91 |  | **hlsp** | **10.47** |
| pc1 | 4432000.00 | 0.02 |  | **h** | **2.16** |
| pa1 | 856600.00 | 0.32 |  | **v** | **1.82** |
| pc2 | 132900000.00 | < 2e-16 |  | **pro_hm** | **0.58** |
| pa2 | 8551000.00 | 0.24 |  | **pc2** | **0.28** |
| ppc | -9640000.00 | 0.18 |  | ac1 | 0.16 |
| ppa | 1138000.00 | 0.16 |  | rho1 | 0.13 |
| x | -72560000.00 | 0.98 |  | m2 | 0.11 |
| Kv | -13.94 | 0.89 |  | ah1 | 0.11 |
| m1 | 33370000.00 | 0.00 |  | pm | 0.09 |
| m2 | 101300000.00 | < 2e-16 |  | tfr1 | 0.07 |
| ps | -15970000.00 | 0.03 |  | m1 | 0.04 |
| pst | -11570000.00 | < 2e-16 |  | pn | 0.02 |
| pn | 538000.00 | 0.46 |  | pc1 | 0.02 |
| pm | -1864000.00 | 0.01 |  | rho2 | 0.01 |
| ss | -3908.00 | 0.89 |  | t1 | 0.01 |
| h | -31590.00 | 0.57 |  | ps | 0.01 |
| q | -15140000.00 | 0.48 |  | ppa | 0.01 |
| gamma | 62860.00 | 0.80 |  | ppc | 0.01 |
| t1 | -16880.00 | 0.06 |  | pa2 | 0.01 |
| rho1 | -14350.00 | 0.12 |  | AC | 0.01 |
| ptq2 | 150200.00 | 0.84 |  | ss | 0.01 |
| rho2 | 93010.00 | 0.02 |  | pa1 | 0.01 |
| v | -74080.00 | 0.03 |  | pro_mh | 0.01 |
| nr | 7937000.00 | 0.41 |  | pst | 0.01 |
| ah1 | 3824000.00 | 0.00 |  | pt3 | 0.01 |
| ah2 | 9822000.00 | 0.50 |  | nr | 0.00 |
| px | -587500.00 | 0.97 |  | Kv | 0.00 |
| pt3 | 1004000.00 | 0.17 |  | ptq2 | 0.00 |
| ah3 | 492200.00 | 0.50 |  | px | 0.00 |
| hlsp | 12430.00 | 0.72 |  | gamma | 0.00 |
| pro_hm | 45690000.00 | < 2e-16 |  | ah2 | 0.00 |
| pro_mh | 53790000.00 | < 2e-16 |  | x | 0.00 |
| AC | 689900.00 | 0.19 |  | ac2 | 0.00 |
| ac1 | 606600.00 | 0.40 |  | ah3 | 0.00 |
| ac2 | 14810000.00 | 0.31 |  | q | 0.00 |
| tfr1 | -2.85E+05 | 0.59 |  |  |  |

**References**

[1] National Malaria Control Program. An epidemiological profile of malaria and its control in Ghana. [Internet]. National Malaria Control Program; 2013 [cited 2016 Feb 15]. Available from: https://www.linkmalaria.org/sites/www.linkmalaria.org/files/content/country/profiles/Ghana-epi-report-2014.pdf.

[2] Awine T, Malm K, Peprah NY, et al. Spatio-temporal heterogeneity of malaria morbidity in Ghana: Analysis of routine health facility data. Munderloh UG, editor. PLOS ONE. 2018;13:e0191707.

[3] Enweronu-Laryea CC, Adjei GO, Mensah B, et al. Prevalence of congenital malaria in high-risk Ghanaian newborns: a cross-sectional study. Malar. J. 2013;12:17.

[4] Unpublished. Preventive treatment of Malaria in Pregnancy, maternally transferred antibodies to malaria and the risk of clinical malaria in a five year Birth cohort in Ghana. 2014.

[5] United States Agency for International Development. Ghana Malaria Operational Plan FY 2017 [Internet]. 2017 [cited 2017 May 11]. Available from: https://www.pmi.gov/docs/default-source/default-document-library/malaria-operational-plans/fy17/fy-2017-ghana-malaria-operational-plan.pdf?sfvrsn=6.

[6] NMCP,Ghana. NMCP Annual Bulletin. 2016.

[7] Falade C, Mokuolu O, Okafor H, et al. Epidemiology of congenital malaria in Nigeria: a multi-centre study: Epidemiology of congenital malaria in Nigeria. Trop. Med. Int. Health. 2007;12:1279–1287.

[8] Ministry of Health,Ghana. Guideline for case managemnet of malaria in Ghana [Internet]. 2014 [cited 2019 Jul 2]. Available from: https://www.ghanahealthservice.org/downloads/GUIDELINE%20FOR%20CASE%20MANAGEMENT%20.pdf.

[9] Lawn JE, Blencowe H, Waiswa P, et al. Stillbirths: rates, risk factors, and acceleration towards 2030. The Lancet. 2016;387:587–603.

[10] Owusu-Boateng I, Anto F. Intermittent preventive treatment of malaria in pregnancy: a cross-sectional survey to assess uptake of the new sulfadoxine–pyrimethamine five dose policy in Ghana. Malar. J. 2017;16:323.

[11] Dinko B, Ayivor R, Abugri J, et al. Comparison of malaria diagnostic methods in four hospitals in the Volta region of Ghana. 2016;7:7.

[12] . World Health Organization (WHO). Implementing Malaria in Pregnancy Programs in the Context of World Health Organization Recommendations on Antenatal Care for a Positive Pregnancy Experience.Geneva, Switzerland: WHO; 2017. Licence: CC BY-NC-SA 3.0 IGO. WHO/RHR/18.05. [Internet]. 2018. Available from: https://apps.who.int/iris/bitstream/handle/10665/259954/WHO-RHR-18.05-eng.pdf?sequence=1.

[13] White NJ, Pukrittayakamee S, Hien TT, et al. Malaria. The Lancet. 2014;383:723–735.

[14] Institute for Health Metrics and Evaluation. Global Health Data Exchange,GBD Compare [Internet]. Available from: https://vizhub.healthdata.org/gbd-compare/#.

[15] White NJ. Malaria parasite clearance. Malar J. 2017;14.

[16] Agusto FB, Gumel AB, Parham PE. QUALITATIVE ASSESSMENT OF THE ROLE OF TEMPERATURE VARIATIONS ON MALARIA TRANSMISSION DYNAMICS. J. Biol. Syst. 2015;23:1550030.

[17] Tran A, L’Ambert G, Lacour G, et al. A Rainfall- and Temperature-Driven Abundance Model for Aedes albopictus Populations. Int. J. Environ. Res. Public. Health. 2013;10:1698–1719.

[18] Oduro AR, Koram KA, Rogers W, et al. Severe falciparum malaria in young children of the Kassena-Nankana district of northern Ghana. Malar. J. 2007;6:96.

[19] Ghana Statistical Service (GSS), Ghana Health Service (GHS). Ghana Malaria Indictor Survey, 2016 [Internet]. Accra, Ghana, and Rockville, Maryland, USA: GSS, GHS, and ICF; 2017. Available from: www.DHSprogram.com.

[20] Mandal S, Sarkar RR, Sinha S. Mathematical models of malaria - a review. Malar. J. 2011;10:202.

[21] Nwaefuna EK, Afoakwah R, Orish VN, et al. Effectiveness of Intermittent Preventive Treatment in Pregnancy with Sulphadoxine-Pyrimethamine against Submicroscopic *falciparum* Malaria in Central Region, Ghana. J. Parasitol. Res. 2015;2015:1–6.

[22] Attakorah J. EVALUATION OF THE LEVEL OF ADHERENCE TO THE ANTIMALARIAL DRUG POLICY BY PRESCRIBERS IN THE TREATMENT OF MALARIA IN CHILD HEALTH DIRECTORATE AT KOMFO ANOKYE TEACHING HOSPITAL. [Internet]. Available from: http://ir.knust.edu.gh/xmlui/bitstream/handle/123456789/843/JOSEPH%20ATTAKORAH.pdf?sequence=1.

[23] Raifman JRG, Lanthorn HE, Rokicki S, et al. The Impact of Text Message Reminders on Adherence to Antimalarial Treatment in Northern Ghana: A Randomized Trial. Slutsker L, editor. PLoS ONE. 2014;9:e109032.

[24] Ampadu HH, Asante KP, Bosomprah S, et al. Prescribing patterns and compliance with World Health Organization recommendations for the management of severe malaria: a modified cohort event monitoring study in public health facilities in Ghana and Uganda. Malar. J. [Internet]. 2019 [cited 2019 Jul 2];18. Available from: https://www.ncbi.nlm.nih.gov/pmc/articles/PMC6368732/.

[25] Abuaku B, Duah-Quashie NO, Quaye L, et al. Therapeutic efficacy of artesunate–amodiaquine and artemether–lumefantrine combinations for uncomplicated malaria in 10 sentinel sites across Ghana: 2015–2017. Malar. J. 2019;18:206.

[26] Taylor TE, Taylor TE, Wills BA, et al. Intramuscular artemether vs intravenous quinine: an open, randomized trial in Malawian children with cerebral malaria. Trop. Med. Int. Health. 1998;3:3–8.

[27] Tagbor H, Bruce J, Ord R, et al. Comparison of the therapeutic efficacy of chloroquine and sulphadoxine-pyremethamine in children and pregnant women. Trop. Med. Int. Health. 2007;12:1288–1297.

[28] Anabire NG, Aryee PA, Abdul-Karim A, et al. Prevalence of malaria and hepatitis B among pregnant women in Northern Ghana: Comparing RDTs with PCR. PLoS ONE [Internet]. 2019 [cited 2019 Jul 24];14. Available from: https://www.ncbi.nlm.nih.gov/pmc/articles/PMC6364880/.

[29] Tay SCK, Nani EA, Walana W. Parasitic infections and maternal anaemia among expectant mothers in the Dangme East District of Ghana. BMC Res. Notes [Internet]. 2017 [cited 2017 Jul 10];10. Available from: http://bmcresnotes.biomedcentral.com/articles/10.1186/s13104-016-2327-5.

[30] Yeboah DF, Afoakwah R, Nwaefuna EK, et al. Quality of Sulfadoxine-Pyrimethamine Given as Antimalarial Prophylaxis in Pregnant Women in Selected Health Facilities in Central Region of Ghana. J. Parasitol. Res. 2016;2016:1–6.

[31] Oduro AR, Wak G, Azongo D, et al. Profile of the Navrongo Health and Demographic Surveillance System. Int. J. Epidemiol. 2012;41:968–976.

[32] Owusu-Agyei S, Nettey OEA, Zandoh C, et al. Demographic patterns and trends in Central Ghana: baseline indicators from the Kintampo Health and Demographic Surveillance System. Glob. Health Action. 2012;5:19033.

[33] Gyapong M, Sarpong D, Awini E, et al. Profile: The Dodowa HDSS. Int. J. Epidemiol. 2013;42:1686–1696.

[34] Kesteman T, Randrianarivelojosia M, Rogier C. The protective effectiveness of control interventions for malaria prevention: a systematic review of the literature. F1000Research. 2017;6:1932.

[35] Mohapatra M. The Natural History of Complicated Falciparum Malaria — A Prospective Study. 2006;54:6.

[36] Appiah MK, Diji AK-A. Rural Folks’ Knowledge on and adherence towards Artemisinin-based combination Therepies. ijird. 2016;5.

[37] Amponsah AO, Vosper H, Marfo AFA. Patient Related Factors Affecting Adherence to Antimalarial Medication in an Urban Estate in Ghana. Malar. Res. Treat. 2015;2015:1–8.

[38] Bartoloni A, Zammarchi L. CLINICAL ASPECTS OF UNCOMPLICATED AND SEVERE MALARIA. Mediterr. J. Hematol. Infect. Dis. 2012;4:2012026.

[39] Makanga M, Krudsood S. The clinical efficacy of artemether/lumefantrine (Coartem). Malar. J. 2009;8 Suppl 1:S5.

[40] Pasvol G. The treatment of complicated and severe malaria. Br. Med. Bull. 2005;75–76:29–47.

[41] Silal SP, Little F, Barnes KI, et al. Predicting the impact of border control on malaria transmission: a simulated focal screen and treat campaign. Malar. J. 2015;14.

[42] Miller MJ. Observations on the natural history of malaria in the semi-resistant West African. Trans. R. Soc. Trop. Med. Hyg. 1958;52:152–168.

[43] WHO Model Prescribing Information: Drugs Used in Parasitic Diseases - Second Edition: Protozoa: Malaria: Pyrimethamine/sulfadoxine [Internet]. [cited 2019 Apr 17]. Available from: http://apps.who.int/medicinedocs/en/d/Jh2922e/2.5.3.html#Jh2922e.2.5.3.

[44] Asoala V. Malaria transmission dynamics and insecticide resistance of malaria vectors in the Kassena-Nankana districts of Ghana. Kwame Nkrumah University of Science and Technology; (Unpublished).

[45] Quartey AA. Estimation of malaria transmission intensity in southern Ghana using rapid diagnostic test derived sero-prevalence rates. [Internet]. Kwame Nkrumah University of Science and Technology; 2016. Available from: http://ir.knust.edu.gh/xmlui/handle/123456789/7048/browse?value=Quartey%2C+Alberta+Amu&type=author.

[46] Ghana Statistical Service and Ghana Health Service. Ghana Demographic and Health Survey 2008 [Internet]. 2009 [cited 2019 Feb 13]. Available from: https://www.dhsprogram.com/pubs/pdf/FR221/FR221[13Aug2012].pdf.

[47] Ghana Statistical Service. Ghana Multiple Indicator Cluster Survey with an Enhanced Malaria Module and Biomaker,2011, Final report. [Internet]. Ghana Statistical Service; 2011 [cited 2015 Dec 23]. Available from: http://www.unicef.org/ghana/Ghana_MICS_Final.pdf.

[48] Ghana Statistical Service. Ghana Demographic and Health Survey,2014 [Internet]. Ghana Statistical Service; 2014 [cited 2015 Dec 23]. Available from: http://dhsprogram.com/what-we-do/survey/survey-display-437.cfm.

[49] Hartig F, Calabrese JM, Reineking B, et al. Statistical inference for stochastic simulation models - theory and application: Inference for stochastic simulation models. Ecol. Lett. 2011;14:816–827.

[50] Hermans B. Application of approximate Bayesian computation to estimate parameters in models of infectious disease spread on a network. :16.

[51] Marjoram P, Molitor J, Plagnol V, et al. Markov chain Monte Carlo without likelihoods. Proc. Natl. Acad. Sci. 2003;100:15324–15328.

[52] Beaumont MA. Approximate Bayesian Computation in Evolution and Ecology. Annu. Rev. Ecol. Evol. Syst. 2010;41:379–406.

[53] van der Vaart E, Beaumont MA, Johnston ASA, et al. Calibration and evaluation of individual-based models using Approximate Bayesian Computation. Ecol. Model. 2015;312:182–190.

[54] Csillery K, Lemaire L, Francois O, et al. Approximate Bayesian Computation (ABC) in R: A Vignette. :24.

[55] Hannah Koenker, Emily Ricotta, Bolanle Olapeju, et al. Insecticide-Treated Nets (ITN) Access and Use Report.Baltimore, MD. PMI | VectorWorks Project, Johns Hopkins Center for Communication Programs. 2018.
